# A Forward Genetic Screen for Molecules Involved in Pheromone-Induced Dauer Formation in *Caenorhabditis elegans*

**DOI:** 10.1534/g3.115.026450

**Published:** 2016-03-10

**Authors:** Scott J. Neal, JiSoo Park, Danielle DiTirro, Jason Yoon, Mayumi Shibuya, Woochan Choi, Frank C. Schroeder, Rebecca A. Butcher, Kyuhyung Kim, Piali Sengupta

**Affiliations:** * Department of Biology, Brandeis University, Waltham, Massachusetts 02454; † National Center for Behavioral Genomics, Brandeis University, Waltham, Massachusetts 02454; ‡ Department of Brain and Cognitive Sciences, DGIST, Daegu 711-873, Republic of Korea; § Boyce Thompson Institute, Cornell University, Ithaca, New York 14853; ** Department of Chemistry and Chemical Biology, Cornell University, Ithaca, New York 14853; †† Department of Chemistry, University of Florida, Gainesville, Florida 32611

**Keywords:** *C. elegans*, dauer, pheromone, *che*-*12*, *maco*-*1*, *qui*-*1*, *ttbk*

## Abstract

Animals must constantly assess their surroundings and integrate sensory cues to make appropriate behavioral and developmental decisions. Pheromones produced by conspecific individuals provide critical information regarding environmental conditions. Ascaroside pheromone concentration and composition are instructive in the decision of *Caenorhabditis elegans* to either develop into a reproductive adult or enter into the stress-resistant alternate dauer developmental stage. Pheromones are sensed by a small set of sensory neurons, and integrated with additional environmental cues, to regulate neuroendocrine signaling and dauer formation. To identify molecules required for pheromone-induced dauer formation, we performed an unbiased forward genetic screen and identified *phd* (pheromone response-defective dauer) mutants. Here, we describe new roles in dauer formation for previously identified neuronal molecules such as the WD40 domain protein QUI-1 and MACO-1 Macoilin, report new roles for nociceptive neurons in modulating pheromone-induced dauer formation, and identify tau tubulin kinases as new genes involved in dauer formation. Thus, *phd* mutants define loci required for the detection, transmission, or integration of pheromone signals in the regulation of dauer formation.

Phenotypic plasticity in response to adverse environmental cues represents a bet-hedging strategy in an unpredictable environment (Avery 2014; Furness *et al.* 2015). A particularly well-studied form of phenotypic plasticity is facultative diapause, a hibernation-like state characterized by cessation of feeding and altered physiology (Denlinger 2002; Emerson *et al.* 2009; Guidetti *et al.* 2011; Schiesari and O’Connor 2013). The decision between entry into diapause or continued reproductive growth is mediated via the integration of environmental cues such as temperature, food, and light to regulate endocrine signaling (Golden and Riddle 1984b; Fielenbach and Antebi 2008; Emerson *et al.* 2009; Hahn and Denlinger 2011; Nylin 2013; Denlinger and Armbruster 2014; Furness *et al.* 2015). Although much is now known about the hormone signaling pathways that regulate diapause, particularly in invertebrates, the neuronal and molecular mechanisms that transduce and integrate sensory cues of multiple modalities to enable this adaptive developmental decision are poorly understood.

*Caenorhabditis elegans* provides an experimentally amenable system in which to dissect the genetic mechanisms that underlie entry into, and exit from, diapause. Shortly after hatching, *C. elegans* larvae assess environmental temperature, availability of food, and population density to choose between the alternate developmental trajectories of reproductive growth, or entry into the dauer diapause-like state (Cassada and Russell 1975; Swanson and Riddle 1981; Golden and Riddle 1982, 1984b). Genetic screens for mutants that enter constitutively into the dauer stage (dauer formation constitutive – Daf-c) or fail to form dauers (dauer formation defective – Daf-d) regardless of environmental conditions, have led to a detailed description of the neuroendocrine signaling pathways that underlie the dauer developmental decision (Albert *et al.* 1981; Riddle *et al.* 1981; Perkins *et al.* 1986; Albert and Riddle 1988; Vowels and Thomas 1992). In brief, environmental cues are integrated to modulate TGF-β and insulin signaling pathways, which act in parallel to regulate steroid hormone signaling (Thomas *et al.* 1993; Riddle and Albert 1997; Hu 2007; Fielenbach and Antebi 2008) (Figure 1A). Under adverse conditions, downregulation of expression of TGF-β and insulin-like peptide (ILP) genes in sensory neurons, such as ASI present in the head amphid organs, results in decreased steroid hormone signaling and dauer entry (Figure 1A). Conversely, under optimal conditions, increased TGF-β and ILP signaling promotes reproductive growth. Consequently, inappropriate modulation of TGF-β, ILP, or steroid hormone signaling results in Daf-d or Daf-c phenotypes.

**Figure 1:**
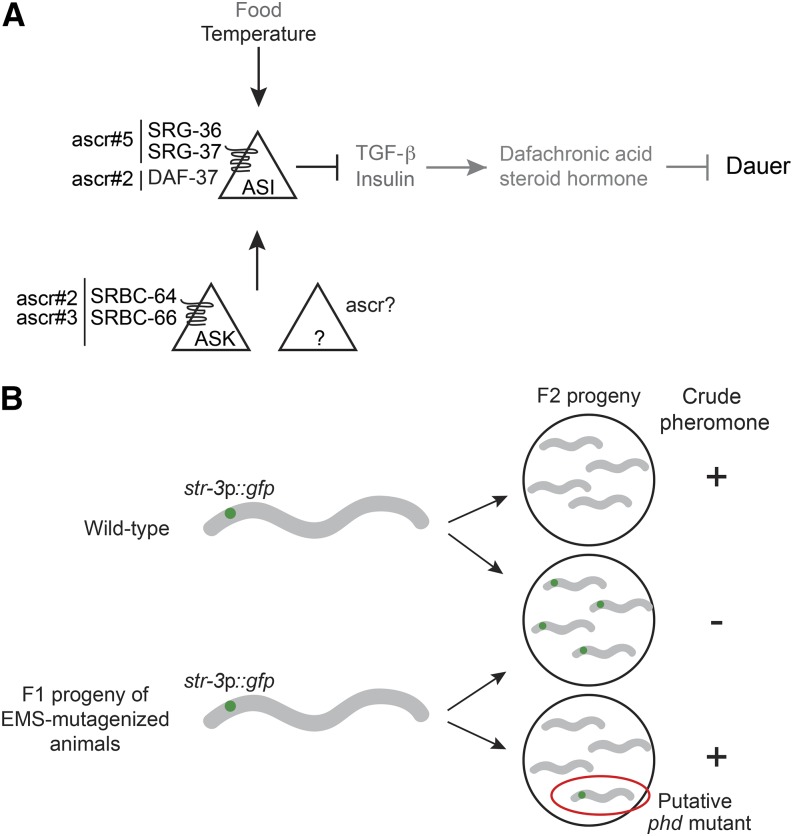
Identification of *phd* mutants. (A) Simplified model of the neuroendocrine signaling pathway regulating dauer formation in response to environmental cues. Ascarosides (ascr) are sensed by G protein-coupled receptors in ASI, in ASK, or possibly in other unidentified sensory neurons. High pheromone concentrations, low food availability, and high temperature cues are integrated to downregulate TGF-β (transforming growth factor-β) and insulin-like peptide gene expression primarily in the ASI sensory neurons. Downregulated steroid hormone signaling in turn promotes dauer formation. (B) Schematic of the forward genetic screen designed to identify putative *phd* mutants based on pheromone-mediated downregulation of *str-3*p::*gfp* expression in the ASI sensory neurons. See text for details. *phd*, pheromone response-defective dauer.

Although the neuroendocrine pathways underlying dauer formation are well described in *C. elegans*, the mechanisms by which environmental cues are sensed and integrated to regulate TGF-β and ILP signaling remain poorly characterized. Within the physiological temperature range, population density as assessed by pheromone concentrations is the instructive environmental trigger for dauer entry, whereas food availability and temperature play permissive roles (Golden and Riddle 1984a,b). *C. elegans* pheromones are now known to be comprised of an unexpectedly large number of small chemicals of diverse structures called ascarosides, derived from the sugar ascarylose (Jeong *et al.* 2005; Butcher *et al.* 2007; Edison 2009; Ludewig and Schroeder 2013). Pheromone signals detected directly by ASI and/or by other chemosensory neurons such as ASK regulate neuroendocrine gene expression in ASI as a function of environmental state (Figure 1A). The ascr#5 (C3, asc-ωC3, daumone 5) and ascr#2 (C6, asc-C6-MK, daumone 2) ascarosides have been shown to be detected by the SRG-36/SRG-37 and DAF-37 G protein-coupled receptors (GPCRs), respectively, acting in the ASI chemosensory neurons (McGrath *et al.* 2011; Park *et al.* 2012), whereas ascr#2 and ascr#3 (C9, asc-ΔC9, daumone 3) are also detected by the SRBC-64/SRBC-66 GPCRs in the ASK chemosensory neurons that regulate dauer formation (Kim *et al.* 2009) (Figure 1A). Sites of detection of other ascarosides in the dauer decision are largely uncharacterized. Moreover, additional signaling molecules required for the transduction of ascaroside signals within ASK or ASI, or for transmission of pheromone information from ASK or other pheromone-sensing neurons to ASI, are unidentified.

We performed an unbiased forward genetic screen to identify molecules required for both intra- and intercellular transduction of pheromone signals. A major goal of this screen was to identify mutants that may exhibit defects in dauer formation in response to subsets of ascarosides. Here, we report the isolation and characterization of pheromone response defective dauer (*phd*) mutants. We identified the causative lesions in a subset of these mutants via whole-genome sequencing and complementation. Our results describe previously uncharacterized roles for the WD40 domain-containing protein QUI-1, the HEAT repeat-containing protein CHE-12, the macoilin protein MACO-1, and tau tubulin kinases in pheromone-regulated dauer formation. Our analyses also define neuronal sites of action of these proteins in pheromone-induced dauer formation. We expect that loci identified in this screen will provide the foundation for additional studies focused on the complex mechanisms by which environmental cues are sensed, weighted, and integrated to direct a binary developmental decision.

## Materials and Methods

### C. elegans strains

*C. elegans* strains were maintained on nematode growth medium (NGM) agar plates at 20°, with *Escherichia coli* OP50 as a food source. The wild-type strain was N2 (Bristol). A list of all strains used in this work is provided in Supplemental Material, Table S1.

### EMS mutagenesis screen

L4 larvae of the strain CX3596 (*kyIs128* [*str-3*p::*gfp*; *lin-15+*]) were mutagenized with ethylmethanesulfonate (EMS) using standard methods (Brenner 1974; Kutscher and Shaham 2014). F1 progeny of mutagenized animals were allowed to lay eggs on plates containing 3 units of crude pheromone (1 unit being defined as the amount necessary to induce 33% dauers on heat-killed OP50 bacteria at 25°). Young adult F2 animals were examined for bright GFP expression in the ASI neurons using a fluorescence dissection microscope. Putative mutants were singled out, and their progeny were retested on plates with and without crude pheromone.

### Dye-filling and dauer formation assays

Animals were filled with DiI as described previously (Perkins *et al.* 1986; Herman and Hedgecock 1990) and examined under a compound microscope. Five pairs of sensory neurons in the head consistently and robustly dye-fill in wild-type animals; the ASI neurons dye-fill more variably and were excluded from this analysis. Mutants exhibiting weak or no dye-filling in at least one of these five head neuron pairs in ≥50% of animals were considered partially dye-fill defective (pDyf). Animals in which all five head sensory neuron pairs exhibited weak or no dye-filling in 50–80% of animals were considered strongly dye-fill defective (sDyf). Mutants that failed to dye-fill all five pairs of head neurons in 80–100% of animals were considered fully dye-fill defective (Dyf).

Dauer formation assays using ascarosides were performed as previously described (Neal *et al.* 2013). Animals were maintained under standard culture conditions at either 20° or 25° for at least three generations prior to being tested for dauer formation at 25°. For dauer formation experiments in the presence of quinine, 3 µL of a 100 mM stock solution of quinine hydrochloride dihydrate (Sigma) was added to the molten agar during the preparation of assay plates.

### Quantification of str-3p::gfp expression

Five well-fed and growth-synchronized adult animals were grown on NGM assay plates containing either ethanol or a pheromone mixture (1 µM each of ascr#2, ascr#3 and ascr#5 or 1 unit crude pheromone) for 4–5 hr until they laid 60–80 eggs. Adult animals were removed, and assay plates were placed at 25° for 60–80 hr until eggs developed into young adults. For GFP quantification, animals were anesthetized using 50 mM sodium azide on an agar pad and were visualized using a Zeiss Axio Imager. All strains were assayed in parallel in two independent experiments.

### Genetic complementation and linkage mapping

To perform complementation testing among isolated alleles, *phd* mutant strains were first injected with a fluorescent marker (*unc-122*p::*dsRed*) to enable identification of cross-progeny. Males from strains carrying the marker as an extrachromosomal array were mated with hermaphrodites from an unmarked strain, and the F2 progeny of marked F1 hermaphrodites from the cross were examined for dauer formation and expression of *str-3*p::*gfp* on pheromone-containing plates. Complementation with wild-type was used to determine whether alleles were dominant or recessive.

To map alleles to linkage groups, we first examined expression of *str-3*p::*gfp* in the F2 progeny of *phd* mutants mated to strains containing recessive mutations on each linkage group leading to visible phenotypes (Fay 2013). We also crossed putative *phd* mutants to strains containing dominant markers on each linkage group, and examined dauer formation in their progeny. We were unable to map using single nucleotide polymorphisms between the N2 Bristol and the Hawaiian CB4856 *C. elegans* isolates (Jakubowski and Kornfeld 1999; Wicks *et al.* 2001; Davis *et al.* 2005), since CB4856 animals were Daf-d under all tested conditions.

### Whole-genome resequencing

Mutants were not outcrossed prior to sequencing. Sequencing libraries from genomic DNA isolated from each *phd* mutant, and sequencing on the Illumina Genome Analyzer IIx platform, were prepared and performed as previously described (Sarin *et al.* 2008). Demultiplexing, alignment and preliminary sequence analysis were performed using the MAQGene analysis pipeline (Bigelow *et al.* 2009). Sequence data were reanalyzed using the CloudMap pipeline and default parameters in the published workflow (Minevich *et al.* 2012). Shared polymorphisms among strains were excluded from analysis (Sarin *et al.* 2010). Polymorphisms unique to each strain that were identified by at least five consensus sequence reads were further followed. Gene models were derived from information in WormBase (www.wormbase.org) and were plotted using WormWeb Tools generated by Nikhil Bhatla (www.wormweb.org).

### Molecular biology

Genomic rescue fragments were amplified from wild-type genomic DNA. Upstream (5′) and downstream (3′) untranslated sequences included in the genomic fragments were the following (relative to the longest predicted isoform in genome release WS220): *che-12*: 837 bp 5′, 100 bp 3′; *maco-1*: 1886 bp 5′, 92 bp 3′; *ttbk-3*: 1109 bp 5′, 242 bp 3′. The fosmid clone encompassing the *qui-1* locus WRM0616aH02 was obtained from Source Bioscience.

cDNA sequences were reverse transcribed from a library generated from the RNA of mixed stage wild-type animals. cDNAs were cloned into the pGEM-T Easy vector (Promega) and were confirmed by sequencing. The *qui-1* cDNA was the generous gift of Paolo Bazzicalupo and Elia di Schiavi, the *che-12* cDNA was the generous gift of Shai Shaham, and the *maco-1* cDNA was the generous gift of Mario de Bono. The following promoters were used for cell-specific expression: ADL (*sre-1*Δp, 1.8 kb), ASH (*sra-6*p, 3.0 kb; also drives weaker expression in ASI), ASI (*srg-47*p, 1.0 kb), ASK (*sra-9*p, 2.9 kb), AWC+ASE (*ceh-36*p, 1.9 kb), and ASJ (*trx-1*p, 1.0 kb). Expression of the *che-12* cDNA was driven by 0.9 kb of *che-12* upstream regulatory sequences.

Transcriptional reporters of *ttbk* gene expression were generated by amplifying upstream regulatory sequences from wild-type genomic DNA (*ttbk-3*, 0.7 kb; *ttbk-4*, 0.4 kb; *ttbk-5*, 0.8 kb; *ttbk-6*, 3.2 kb; *ttbk-7*, 3.0 kb) and fusing them with a *gfp* expression cassette by either overlap extension PCR or by cloning into an expression vector. GFP-tagged *ttbk-3* and *ttbk-4* cDNA sequences were generated by overlap extension PCR to mutate the stop codons and fuse GFP in frame. The *qui-1* cDNA was fused in frame with a four amino acid linker and an artificial intron-containing *mCherry* reporter gene. Constructs were confirmed by sequencing.

### Microscopy

L1 larvae were dye-filled with DiI in M9 buffer and were imaged on a Zeiss Axio Imager.M2 microscope using a 63 × (NA 1.4) oil objective. Larvae were mounted on 2% agarose pads on a microscope slide and were immobilized in 10 mM levamisole (Sigma). Z-stacks (0.25–0.5 µm per slice) were acquired using a Hamamatsu Orca camera. Image analysis and cell identification was performed using Zen Pro (Zeiss) and FIJI (NIH) imaging software.

For cilia length measurements, 1-day-old adult worms were transferred to a 2% agarose pad on a microscope slide and immobilized using 10 mM levamisole. Animals were visualized and images were captured as above. Cilia were measured using the segmented line tool in FIJI imaging software. Expression of *daf-7*p::*gfp(ksIs2)* was examined using a 63 × oil objective. All measurements were performed blind to the genotype.

### Statement on data and reagent availability

All reagents including strains and DNA constructs are freely available upon request. Whole-genome sequencing data have been deposited at the NCBI Sequence Read Archive (accession PRJNA314001; http://www.ncbi.nlm.nih.gov/bioproject/314001).

## Results and Discussion

### Rationale for screen design

Quantitative dauer formation assays under controlled conditions are laborious, and are not readily amenable to high throughput screening (Neal *et al.* 2013). We and others previously showed that ASI-specific expression of a GFP reporter, driven under the regulatory sequences of the *str-3* GPCR gene, is strongly downregulated by pheromones (Peckol *et al.* 2001; Nolan *et al.* 2002; Kim *et al.* 2009) (Figure 1B), and that this downregulation is decreased in animals that are mutant for the ASK-expressed *srbc-64/srbc-66* pheromone receptor genes (Kim *et al.* 2009). Therefore, we reasoned that screening for mutants unable to downregulate *str-3*p::*gfp* expression upon pheromone exposure may allow us to identify mutants defective in pheromone sensation, and/or signal transmission, in the context of dauer formation.

Following mutagenesis of *str-3*p::*gfp*-expressing parent animals by ethyl methanesulfonate (EMS), we screened F2 progeny representing approximately 38,500 haploid genomes for their ability to repress *str-3*p::*gfp* expression when grown on plates containing crude pheromone and plentiful food (see *Materials and Methods*) (Figure 1B). This screen allowed us to identify 129 *phd* mutants that continued to express *str-3*p::*gfp* upon pheromone exposure. Preliminary data from this screen have been reported previously (Kim *et al.* 2009).

### Initial characterization of phd mutants

We first determined whether the isolated mutants were Daf-c or Daf-d. Four of the 129 mutants were Daf-c and were not examined further. Many Daf-d mutants exhibit ciliary or dendritic structural defects in the chemosensory neurons of the head amphid or tail phasmid organs (Thomas *et al.* 1993; Vowels and Thomas 1994); these defects are readily detected due to the inability of a subset of these sensory neurons, including ASK and ASI, to fill with lipophilic dyes such as DiI (Perkins *et al.* 1986; Herman and Hedgecock 1990; Starich *et al.* 1995). Thus, dye-filling serves as a convenient initial screen for animals with such morphological abnormalities. Of the mutants, 58% (75/129) exhibited either complete or nearly complete defects in dye-filling and were not considered further. Of the remaining mutants, we analyzed 26 strains that were sufficiently healthy and fertile to permit quantification of dauer formation.

We further analyzed the dye-filling, pheromone-mediated repression of *str-3*p::*gfp* expression in ASI, and dauer formation phenotypes of the selected mutants. We also included a mutant (*oy141*) that failed to dye-fill for comparison purposes in these assays. Nineteen of these strains exhibited wild-type dye-filling in young adult animals (Table 1), suggesting that the ASK and ASI pheromone-sensing neurons are likely to be generated and correctly specified in these mutants. Four and three strains exhibited partial and more severe defects in dye-filling, respectively (Table 1). As expected, the ability of a mixture of ascr#2, ascr#3 and ascr#5 (1 μm each) to repress *str-3*p::*gfp* expression in ASI was strongly affected in the *srbc-64*; *srbc-66*, and *srg-36srg-37* pheromone receptor mutants, and was compromised in the majority of the examined mutant strains to varying extents (Table 1). The extent of the gene expression defect was not fully correlated with the dye-fill phenotypes of these mutants (Table 1). Thus, a subset of mutants with strong defects in pheromone-mediated *str-3*p::*gfp* repression exhibited wild-type dye-filling (*oy109*, *oy134*; Table 1) and conversely, mutants with dye-fill defects downregulated *str-3*p::*gfp* expression in the presence of pheromone (*oy106*, *oy127*; Table 1). All mutants, with the exception of *oy143*, also exhibited strong defects in dauer formation in response to two concentrations of ascr#2, regardless of their dye-fill or *str-3*p::*gfp* expression phenotype (Table 1). Although the different phenotypes of these *phd* mutants could arise from mutations in distinct loci in these nonoutcrossed strains, these results nevertheless indicate that pheromone-dependent repression of *str-3*p::*gfp* is a useful screening tool for the identification of mutants exhibiting defects in pheromone-regulated dauer formation.

**Table 1:** Initial phenotypic characterization of *phd* mutants

Strain*^a^*	Visible Phenotypes*^b^*	Dye-Filling*^c^*	*str-3*p::*gfp* Expression Index*^d^*	Proportion of Dauers Formed on:*^e^*		
				ascr#2 (nM)		
				0*^f^*	60	600
WT		WT	n/a	0 ± 0	0.4 ± 0.1	0.7 ± 0.1
*str-3*p::*gfp*		WT	0.35	0 ± 0	0.5 ± 0.1	0.6 ± 0.1
*srbc-64*; *srbc-66*	Adult egg production delayed	WT	0.87	0 ± 0	0.1 ± 0.0	0.1 ± 0.1
*srg-36 srg-37*		n/d	0.82	n/d	n/d	n/d
*oy103*	Inviable eggs (25°, crude pheromone)	WT	0.57	0 ± 0	0 ± 0	0.1 ± 0.0
*oy104*		WT	0.59	0 ± 0	0.1 ± 0.1	0.4 ± 0.1
*oy105*	Social, eggs laid at edge of plate	WT	0.53	0.1 ± 0.0	0.1 ± 0.1	0.2 ± 0.1
*oy106*		*pDyf*	0.44	0 ± 0	0 ± 0	0.1 ± 0.0
*oy107*	*egl*, slow development	WT	0.52	0 ± 0	0.2 ± 0.1	0.3 ± 0.1
*oy108*		WT	0.62	0 ± 0	0.1 ± 0.1	0.2 ± 0.1
*oy109*	*egl*, slow development	WT	0.93	0.1 ± 0.0	0 ± 0	0 ± 0
*oy113*		*pDyf*	0.62	0 ± 0	0 ± 0	0 ± 0
*oy117*		WT	0.65	0 ± 0	0 ± 0	0.2 ± 0.1
*oy118*		WT	0.56	0 ± 0	0 ± 0	0.1 ± 0.0
*oy119*		WT	0.42	0 ± 0	0 ± 0.1	0.3 ± 0.1
*oy120*		WT	0.66	0 ± 0	0 ± 0	0 ± 0
*oy125*	*unc*	WT	0.60	0 ± 0	0.1 ± 0.0	0.1 ± 0.1
*oy126*		WT	0.46	0 ± 0	0.1 ± 0.0	0.1 ± 0.1
*oy127*	*egl*, slow development	*sDyf*	0.44	0 ± 0	0 ± 0	0.1 ± 0.0
*oy129*	*unc*	*pDyf*	0.68	0 ± 0	0 ± 0	0 ± 0
*oy131*	*egl*, slow development	*pDyf*	0.55	0 ± 0	0 ± 0	0 ± 0
*oy134*	Asynchronous growth	WT	0.93	0 ± 0	0 ± 0	0 ± 0
*oy135*	Adult egg production delayed	WT	0.71	0 ± 0	0 ± 0	0.1 ± 0.0
*oy137*	Slow development	*sDyf*	0.74	0 ± 0	0 ± 0	0 ± 0
*oy138*	*unc*	WT	0.47	0 ± 0	0 ± 0	0.1 ± 0.1
*oy139*	*lon*, *rol*, thin body size	WT	0.54	0 ± 0	0 ± 0	0 ± 0
*oy140*		WT	0.56	0 ± 0	0 ± 0	0.1 ± 0.0
*oy141*		*Dyf*	0.95	0 ± 0	0 ± 0	0 ± 0
*oy142*		*sDyf*	0.85	0 ± 0	0 ± 0	0 ± 0
*oy143*		WT	0.57	0 ± 0	0.3 ± 0.1	0.6 ± 0.1
*oy144*	Inviable eggs (25°, crude pheromone)	WT	n/d	0 ± 0	0 ± 0	0.1 ± 0.0

ascr, ascaroside; WT, wild-type; n/a, not applicable; n/d, not done; *egl*, delayed egg-laying; p*Dyf*, partially dye-fill defective; *unc*, uncoordinated; s*Dyf*, strongly dye-fill defective; *lon*, long; rol rollers; *Dyf*, fully dye-fill defective.

a All strains, except WT, contain stably integrated copies of the *str-3*p::*gfp* fusion gene (*kyIs128*).

b Phenotypes are reported only if they are observed in > 50% of animals. Social indicates that animals aggregate.

c Animals were filled with DiI and amphid neurons were visualized. Criteria for classification as WT, p*Dyf*, s*Dyf*, and *Dyf* are described in *Materials and Methods*.

d *str-3*p::*gfp* expression was observed in animals grown in the presence or absence of 1 µM each ascr#2, ascr#3, and ascr#5 at 25°, and a subjective expression value with arbitrary values of 0–10 was assigned to each animal. The index presented is the ratio of expression in animals grown on pheromone, divided by the expression in control animals. *n* = 30 animals/condition/trial; at least two independent trials.

e Numbers shown are the proportion of dauers formed in the given condition from two (0 nM) or three (60 nM, 600 nM) independent experiments at 25° with 40–110 animals per assay. Two technical replicates were performed in each experiment. Errors are SEM (standard errors of the mean).

f Plates contained 6 µL of ethanol which was used as the diluent for ascr#2.

### phd mutants exhibit defects in dauer formation in response to specific ascarosides

**Figure 2:**
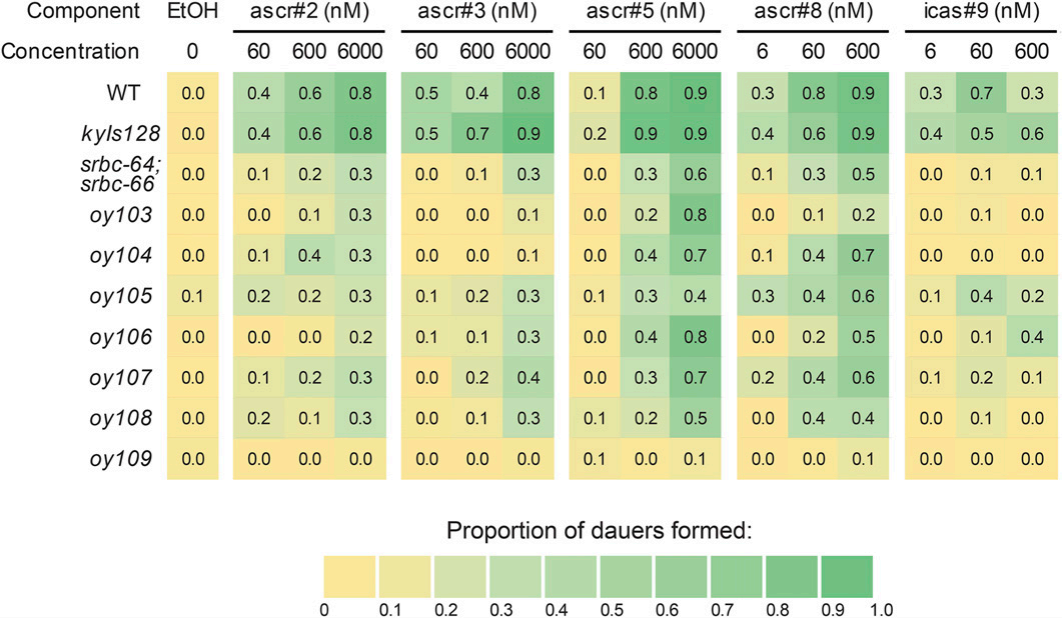
Dauer formation defects exhibited by *phd* mutants in response to different ascarosides. Shown are the proportions of dauers formed by strains of the indicated genotypes in response to different concentrations of ascr#2, ascr#3, ascr#5, ascr#8, and icas#9 at 25°. Numbers shown are the average from three biologically independent assays of two technical replicates each. Averages with SEM (standard error of the mean) are shown in Table S2. All strains with the exception of wild-type (WT) and *srbc-64*; *srbc-66* contain stably integrated copies of the *str-3*p::*gfp* transgene (*kyIs128*). The *srbc-64(tm1946)* and *srbc-66(tm2943)* alleles were used. ascr, ascarisode; EtOH, ethanol; *phd*, pheromone response-defective dauer.

Given the strong defects in ascr#2-induced dauer formation in the majority of examined *phd* mutants, we next asked whether these mutants exhibit similarly strong defects in dauer formation in response to additional ascarosides. We selected seven *phd* mutants (*oy103–oy109*), which exhibited a range of defects in pheromone-mediated repression of *str-3*p::*gfp* expression, and examined dauer formation in response to multiple concentrations of ascr#3, ascr#5, ascr#8 (asc-ΔC7-PABA), and icas#9 (C5, IC-asc-C5), in addition to ascr#2 (Figure 2 and Table S2).

#### oy103:

*oy103* mutants exhibited strong defects in dauer formation in response to all concentrations of ascr#2, ascr#3, ascr#8, and icas#9. However, *oy103* mutants retained the ability to form dauers in response to high concentrations of ascr#5 (Figure 2 and Table S2).

#### oy104:

*oy104* mutants were strongly defective in dauer formation in response to all examined concentrations of ascr#3 and icas#9, and less defective to ascr#2. Dauer formation in response to ascr#5 and ascr#8 was only weakly affected (Figure 2 and Table S2). Since responses to ascr#2, ascr#3, and icas#9 require ASK (Kim *et al.* 2009) (S. J. Neal and P. Sengupta, unpublished results), *oy104* may be a mutation in a molecule required for ascaroside-specific signal transduction within ASK, between ASK and ASI, or within ASI.

#### oy105:

*oy105* mutants exhibited an overall decrease in dauer formation in response to all concentrations of examined ascarosides (Figure 2 and Table S2), suggesting a generalized defect, or dampening, of pheromone-induced dauer formation in this mutant background.

#### oy106:

*oy106* mutants exhibited defects in dauer formation in response to all examined concentrations of ascr#2 and ascr#3, and strong defects in response to lower concentrations of ascr#8 and icas#9 (Figure 2 and Table S2). Weak defects were also observed in response to low ascr#5 concentrations (Figure 2 and Table S2).

#### oy107:

*oy107* animals exhibited strong defects in dauer formation in response to all concentrations of icas#9, and weaker defects in response to all other examined ascarosides, including low concentrations of ascr#5 (Figure 2 and Table S2).

#### oy108:

Dauer formation defects in *oy108* mutants resembled those of *oy105*, with the exception that these mutants failed to form dauers in response to any concentration of icas#9 (Figure 2 and Table S2).

#### oy109:

Unlike the other examined *phd* mutants, *oy109* mutants exhibited strong defects in dauer formation in response to all concentrations of all examined ascarosides (Figure 2 and Table S2). This mutant also exhibited strong defects in pheromone-mediated repression of *str-3*p::*gfp* expression, similar to *oy141* and *oy142* mutants (Table 1). However, in contrast to the strong dye-fill defects exhibited by *oy141* and *oy142* mutants, dye-filling was unaffected in *oy109* animals (Table 1). Thus, *oy109* may represent a mutation in a gene required for the specific transduction of ascaroside signals.

In summary, we identified mutants that exhibit dauer formation defects in response to specific ascarosides, particularly at low concentrations (*oy104*, *oy106*, and *oy107*), a mutant (*oy103*) which is defective in dauer formation in response to all ascarosides with the exception of high concentrations of ascr#5, a mutant (*oy105*) that exhibits generally decreased dauer formation regardless of ascaroside identity, and a mutant (*oy109*) that fails to form dauers at any concentrations of all examined ascarosides.

### Genetic and molecular analyses of phd genes

We attempted to map each allele to candidate linkage groups based on both their pheromone-induced dauer and *str-3*p::*gfp* expression phenotypes (see *Materials and Methods*). Genetic mapping and complementation analyses suggested that the *oy103–oy109* alleles described above represent recessive mutations in complementing genes (data not shown). Alleles were tentatively mapped to the following linkage groups: *oy103* II, *oy104* I, *oy105* IV, o*y106* V, *oy107* IV, *oy108* I, and *oy109* II. To identify the causal mutations, we sequenced the genomes of these *phd* mutants and identified unique variants in each strain (Table S3). We focused on nonsense mutations in genes located on candidate linkage groups associated with each allele, and performed rescue experiments with wild-type sequences of each candidate gene. Although we were unable to rescue the mutant phenotypes of *oy103*, *oy104* and *oy109*, as described below, we identified genes affected by the *oy105*, *oy106*, *oy107*, and *oy108* mutations.

### oy106 is a mutation in the che-12 ciliary gene

**Figure 3:**
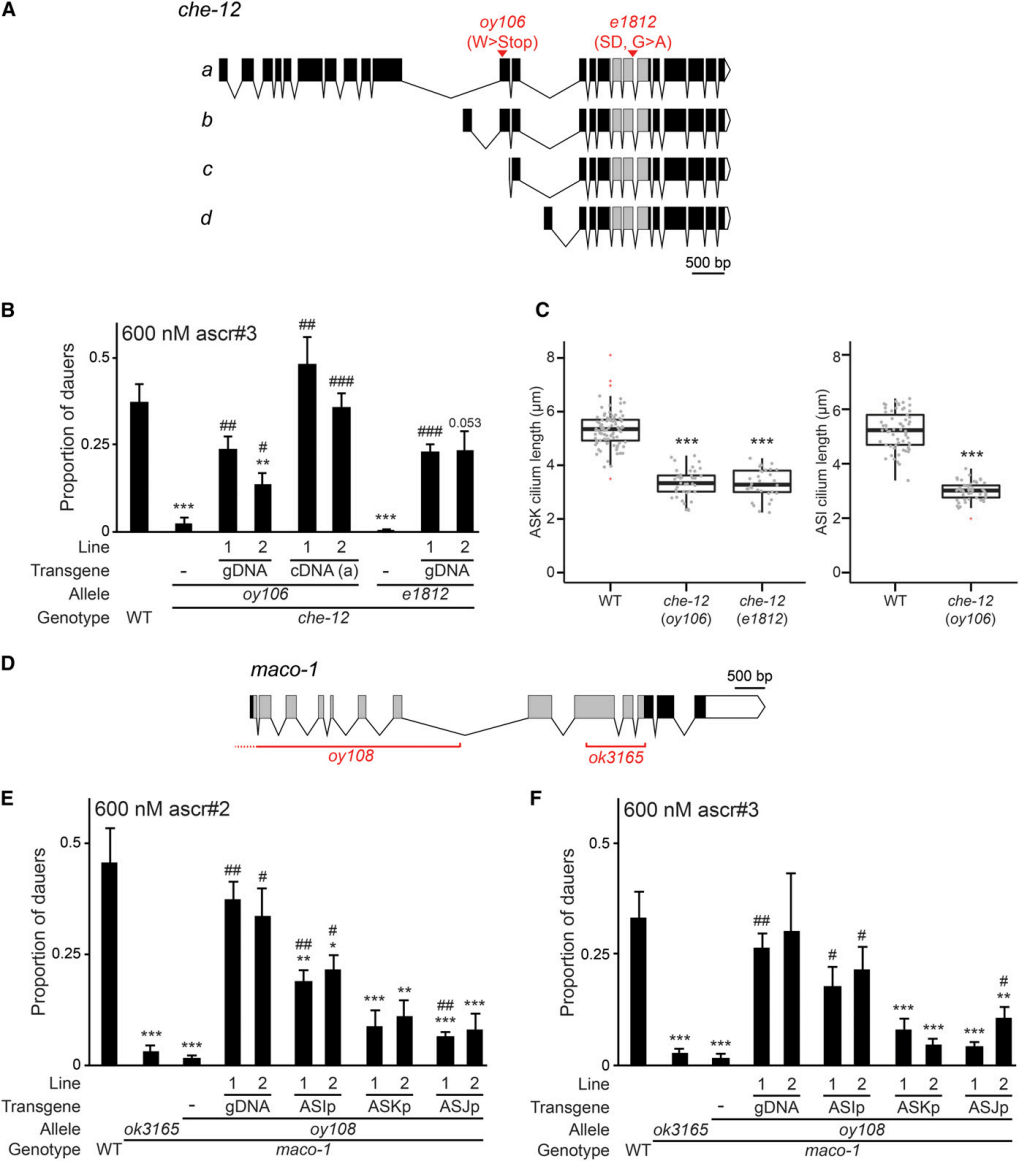
The CHE-12 HEAT domain and MACO-1 macoilin proteins regulate pheromone-induced dauer formation. (A) Predicted exon/intron structures of *che-12* isoforms (a–d). Gray boxes indicate exons predicted to encode HEAT repeats, white triangles indicate 3′ UTRs. The locations of mutations in the *oy106* and *e1812* alleles are indicated. (B) Dauers formed by animals of the indicated genotypes in response to 600 nM ascr#3 at 25°. Lines refer to independent transgenic strains carrying the indicated transgenes on extrachromosomal arrays. The che-12, a cDNA isoform was expressed under 0.9 kb *che-12* upstream regulatory sequences (Bacaj *et al.* 2008). Each data point is the average of at least three biologically independent assays of 40–110 animals each. Errors are SEM. ** and *** indicate different from wild-type at *P* < 0.01 and 0.001, respectively; #, ##, and ### indicate different from *che-12(oy106)* or *che-12(e1812)* at *P* < 0.05, 0.01, and 0.001, respectively (ANOVA and Games-Howell posthoc test). (C) Lengths of ASK (left) and ASI (right) cilia in animals of the indicated genotypes. Cilia were visualized via expression of *srbc-66*p::*mCherry* (ASK) and *str-3*p::*gfp* (ASI). Top and bottom bounds of boxes indicate 25th and 75th percentiles, respectively. Medians are indicated by thick horizontal bars. *** indicates different at *P* < 0.001 from wild-type (*t*-test). Outliers are indicated by red + symbols. (D) Predicted exon/intron structure of the *maco-1* genomic locus. Gray boxes indicate exons encoding the macoilin domain, white triangle indicates 3′ UTR. The extent of the deletions in *maco-1* alleles is indicated. (E and F) Dauers formed by animals of the indicated genotypes in response to 600 nM ascr#2 (E) or 600 nM ascr#3 (F) at 25°. Lines refer to independent transgenic strains carrying the indicated transgenes on extrachromosomal arrays. The *maco-1* cDNA was expressed under *srg-47* (ASI), *sra-9* (ASK), or *trx-1* (ASJ) promoters. Each data point is the average of at least three biologically independent assays of 40–110 animals each. Errors are SEM.*, **, and *** indicate different from wild-type at *P* < 0.05, 0.01, and 0.001, respectively; #, ##, and ### indicate different from *maco-1(oy108)* at *P* < 0.05, 0.01, and 0.001, respectively (ANOVA and Games-Howell posthoc test). ascr, ascaroside; cDNA, complementary DNA; gDNA, genomic DNA; SD, splice donor; SEM, standard error of the mean; UTR, untranslated region; WT, wild-type.

The *oy106* strain contains a premature STOP codon in the *che-12* gene (Figure 3A) (Perkins *et al.* 1986; Starich *et al.* 1995; Bacaj *et al.* 2008) that is predicted to affect the two longer *che-12* isoforms (Figure 3A). The independently isolated *che-12(e1812)* mutant also exhibited strong defects in ascaroside-induced dauer formation (Figure 3B). ascr#3-induced dauer formation defects in *oy106* and *che-12(e1812)* mutants were significantly rescued by wild-type genomic *che-12* sequences, as well as a *che-12* cDNA expressed under *che-12* upstream regulatory sequences (Figure 3B). We conclude that *oy106* is an allele of *che-12*.

*che-12* encodes a HEAT repeat-containing protein belonging to the tubulin-binding Crescerin1 family (Bacaj *et al.* 2008; Das *et al.* 2015). *che-12* is expressed in a subset of amphid sensory neurons, including ASK and ASI, that exhibit simple, rod-like ciliated sensory endings (Perkins *et al.* 1986; Bacaj *et al.* 2008; Doroquez *et al.* 2014; Das *et al.* 2015). CHE-12 is localized to cilia, and ciliary ultrastructure is disrupted in *che-12* mutants (Bacaj *et al.* 2008; Das *et al.* 2015). Cilia house sensory signal transduction molecules and are formed by the process of intraflagellar transport (IFT), which is required for the movement of proteins within cilia (Rosenbaum and Witman 2002; Pedersen and Rosenbaum 2008). Previous studies have indicated that CHE-12 is unlikely to be a component of the IFT complex but requires IFT for ciliary localization (Perkins *et al.* 1986; Bacaj *et al.* 2008). Based on these observations and the roles of other CHE-12 family members, CHE-12 has recently been proposed to be a microtubule-binding and polymerizing protein that is required for correct ciliogenesis and cilia structure maintenance (Bacaj *et al.* 2008; Das *et al.* 2015).

We confirmed that ASK cilia length is significantly shorter in both *che-12* alleles (Figure 3C). In addition, ASI cilia length was also similarly decreased in *che-12(oy106)* mutants (Figure 3C). We conclude that the described role of CHE-12 in regulating the formation and maintenance of cilia in neurons such as ASK and ASI is consistent with the observed pheromone-induced dauer formation defects upon loss of *che-12* function.

### oy108 is a mutation in the maco-1 Macoilin gene

We noted a large deletion in the *oy108* carrying strain that affected two genes including the *maco-1* macoilin locus (Figure 3D). The expression of wildtype genomic *maco-1* sequences was sufficient to rescue dauer formation in *oy108* in response to both ascr#2 and ascr#3 (Figure 3, E and F). Moreover, animals carrying the independently isolated *maco-1(ok3165)* putative null allele also exhibited dauer formation defects similar to those exhibited by *oy108* animals (Figure 3, E and F). Thus, *oy108* affects *maco-1* function.

MACO-1 is expressed broadly in the *C. elegans* nervous system, and is localized to the rough endoplasmic reticulum (Arellano-Carbajal *et al.* 2011; Miyara *et al.* 2011). *maco-1* mutants exhibit pleiotropic neuronal defects, and this protein has been suggested to play a role in the trafficking of transmembrane proteins that regulate neuronal excitability (Arellano-Carbajal *et al.* 2011; Miyara *et al.* 2011). We examined whether MACO-1 acts in specific sensory neuron types to regulate dauer formation. We found that expression of wild-type *maco-1* sequences in ASI rescued both ascr#2- and ascr#3-regulated dauer formation defects of *maco-1(oy108)* mutants more strongly than expression in either the ASK or ASJ sensory neurons (Figure 3, E and F). Expression of the *daf-7* TGF-β ligand in ASI (Ren *et al.* 1996; Schackwitz *et al.* 1996) was unaffected upon loss of *maco-1* function [Figure S1; 100% of wild-type and *maco-1(ok3165)* animals expressed *daf-7*p::*gfp* in ASI; *n* = 20 each]. Since *maco-1* mutants retained the ability to form dauers in response to ascr#5 (Figure 2 and Table S1), we propose that MACO-1 may play a role in the trafficking of as yet unidentified proteins required for transducing ascaroside-specific signals in ASI to regulate dauer formation.

### The QUI-1 WD40 repeat-containing protein acts in nociceptive neurons to facilitate pheromone-induced dauer formation in the presence of noxious chemicals

**Figure 4:**
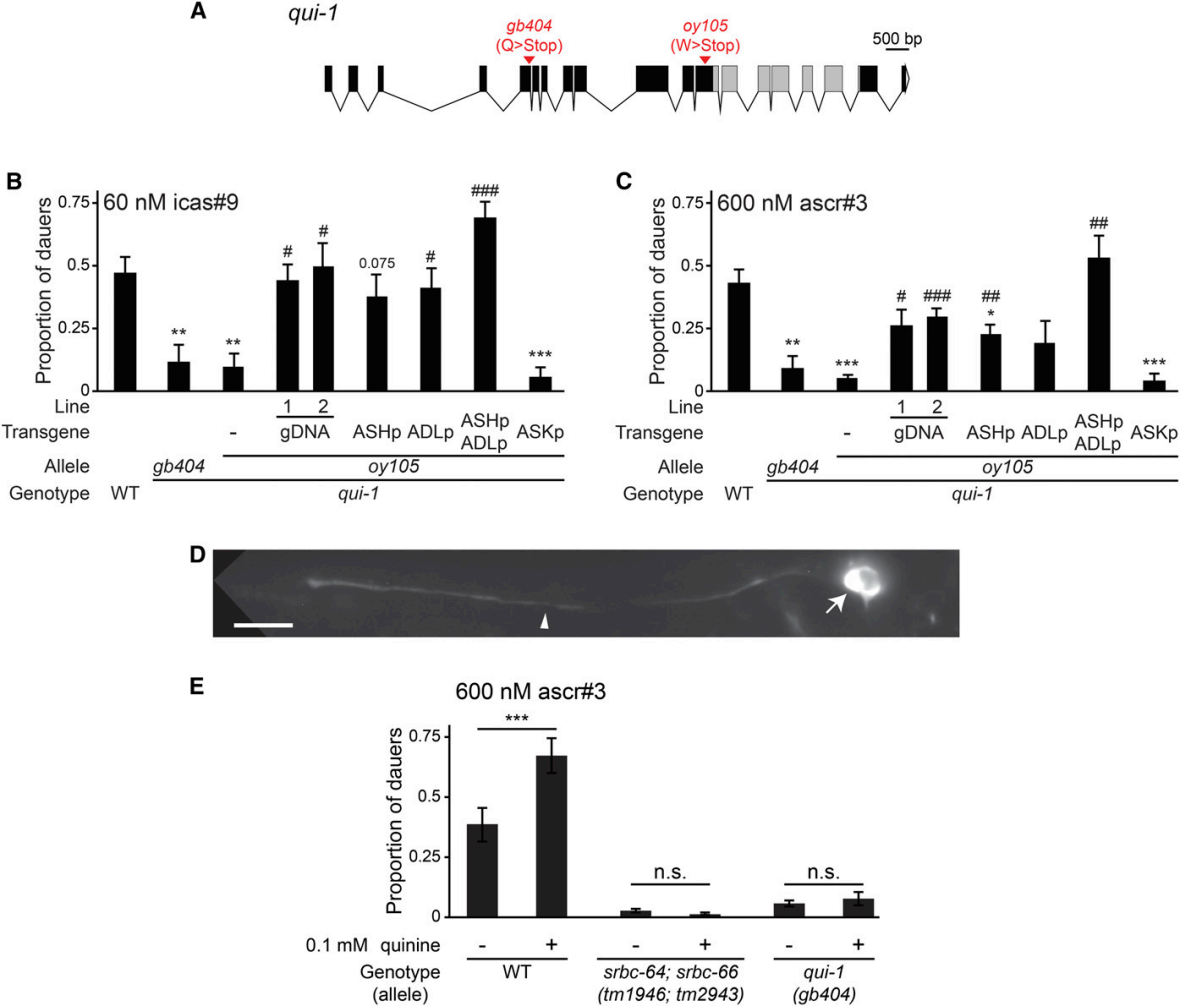
QUI-1 acts in the ASH and ADL nociceptive neurons to increase pheromone-induced dauer formation by noxious chemicals. (A) Predicted exon/intron structure of the *qui-1* genomic locus. Gray boxes indicate exons predicted to encode WD40 repeats, white triangle indicates 3′ UTR. The location and nature of the mutations in *qui-1* alleles are indicated. (B and C) Dauers formed by animals of the indicated genotypes in response to 60 nM icas#9 (B) or 600 nM ascr#3 (C) at 25°. Lines refer to independent transgenic strains carrying the indicated transgenes on extrachromosomal arrays. The *qui-1* cDNA was expressed under the *sra-9* (ASK), *sra-6* (ASH, also drives expression weakly and variably in ASI), *sre-1*Δ*p* (ADL), or both (ASH/ASI/ADL) promoters. Each data point is the average of at least three biologically independent assays of 40–110 animals each. Errors are SEM.*, **, and *** indicate different from wild-type at *P* < 0.05, 0.01, and 0.001, respectively; #, ##, and ### indicate different from *qui-1(oy105)* at *P* < 0.05, 0.01, and 0.001, respectively (ANOVA and Games-Howell posthoc test). (D) Representative image of the localization of QUI-1::mCherry in ASH in an adult hermaphrodite. Arrow and arrowhead indicate cell soma and dendrite, respectively. The bright fluorophore signal in the soma likely represents aggregation due to overexpression. Note exclusion from the nucleus. The fusion protein was also detected weakly in the cilia. Anterior is at left. Scale bar: 10 μm. (E) Dauers formed by animals of the indicated genotypes in response to 600 nM ascr#3 and 0.1 mM quinine at 25°. Each data point is the average of three biologically independent assays of 40–110 animals each. Errors are SEM. For the indicated pairwise comparisons, *** indicates *P* < 0.001 and n.s. indicates not statistically significant (two-tailed *t*-test). ascr, ascaroside; cDNA, complementary DNA; gDNA, genomic DNA; SEM, standard error of the mean; UTR, untranslated region; WT, wild-type.

The *oy105* strain contains a premature termination codon in the predicted coding region of the *qui-1* gene (Figure 4A) (Hilliard *et al.* 2004). We obtained significant rescue of both icas#9- and ascr#3-induced dauer formation defects of *oy105* mutants with a fosmid containing *qui-1* genomic sequences (Figure 4, B and C). The independently isolated *qui-1(gb404)* mutant (Hilliard *et al.* 2004) (Figure 4A) also exhibited dauer formation defects that were qualitatively similar to those of *oy105* mutants (Figure 4, B and C). Together with additional observations described below, these results indicate that *oy105* is an allele of *qui-1*.

*qui-1* alleles were originally identified in a genetic screen for mutants defective in their ability to avoid a subset of aqueous repellents including quinine, as well as low pH (Hilliard *et al.* 2004). *qui-1* encodes a large protein of undefined function, containing multiple WD40 repeats implicated in protein-protein interactions (Figure 4A) (Hilliard *et al.* 2004; Xu and Min 2011; Zhang and Zhang 2015). *qui-1* was shown to be expressed in multiple sensory and nonsensory neurons including in the ASH and ADL nociceptive chemosensory neurons (Hilliard *et al.* 2004); the ASH neurons have previously been shown to respond to bitter compounds such as quinine (Hilliard *et al.* 2005). A GFP-tagged QUI-1 protein localized to the cytoplasm and was excluded from the nuclei of many, but not all, expressing cells (Hilliard *et al.* 2004). A role for QUI-1 in dauer formation has not previously been described.

To first determine where QUI-1 acts to regulate dauer formation, we performed cell-specific rescue experiments. Intriguingly, we found that expression of wild-type *qui-1* sequences in either the ASH or ADL nociceptive neurons partially rescued the dauer formation defects of *qui-1(oy105)* mutants, whereas expression in both neuron types fully rescued dauer formation in this mutant background (Figure 4B). *qui-1* was reported to not be expressed in ASK (Hilliard *et al.* 2004), and consistent with this observation, expression of *qui-1* in ASK failed to rescue (Figure 4B). We reexamined the subcellular localization of QUI-1 in ASH by expressing a mCherry-tagged QUI-1 fusion protein under a cell-specific promoter. QUI-1::mCherry was localized to the ASH cytosol but excluded from nuclei (Figure 4D).

The general decrease in dauer formation in *qui-1* mutants in response to multiple ascarosides implies that QUI-1 may regulate a shared aspect of dauer formation such as modulation of neuroendocrine signaling, possibly via regulation of general pheromone responsiveness. Neither ASH nor ADL have previously been implicated in dauer formation, although ADL mediates aversion to ascr#3 in adult hermaphrodites (Jang *et al.* 2012). Since ascr#3-induced dauer formation is largely mediated by ASK, we considered the hypothesis that QUI-1 acts in ASH and ADL to modulate ASK-mediated pheromone signaling. ASH and ADL have been previously suggested to respond to aversive food stimuli to promote adult aggregation behavior, which is in part mediated by pheromone signaling (White *et al.* 1986; De Bono *et al.* 2002; Macosko *et al.* 2009; Jang *et al.* 2012). Along with ASK, ASH and ADL are present in a hub-and-spoke network motif in which spoke sensory neurons are connected to the RMG hub motor/interneuron via gap junctions (Macosko *et al.* 2009; Jang *et al.* 2012). Moreover, ADL, ASH, and ASK are connected via chemical synapses in a feedforward circuit with ADL being the most upstream, and ASK being the most downstream, neurons in the circuit (White *et al.* 1986). Thus, it is plausible that ASH/ADL activity modulates ASK pheromone signaling.

Since both ASH and ADL respond to noxious stimuli (Kaplan and Horvitz 1993; Hart *et al.* 1995; Maricq *et al.* 1995; Troemel *et al.* 1997; Sambongi *et al.* 1999; Hilliard *et al.* 2002, 2004, 2005), we asked whether activation of one or both of these neurons by noxious chemicals promotes dauer formation, and whether this facilitation is decreased in *qui-1* mutants. To address this issue, we tested whether quinine sensed by ASH (Hilliard *et al.* 2005) enhances pheromone-induced dauer formation. We found that the addition of 0.1 mM quinine enhanced dauer formation in response to ascr#3 in wild-type animals (Figure 4E). Importantly, this enhancement was abolished not only in *qui-1(gb404)* mutants, but also in animals mutant for the *srbc-64* and *srbc-66* ascr#3 receptor genes that are expressed specifically in ASK (Figure 4E). A simple interpretation of these results is that QUI-1-dependent activation of ASH or ADL by noxious chemicals, possibly from food, facilitates pheromone signaling from ASK to result in increased dauer formation. This facilitation could occur at the level of pheromone responsiveness or pheromone signal output from ASK. ASH and ASI have recently been shown to reciprocally inhibit each other in the context of adult nociceptive behavior (Guo *et al.* 2015). Thus, QUI-1 activity in ASH/ADL may also modulate pheromone responsiveness in ASI to regulate dauer formation. While the precise cellular functions of QUI-1 in sensory signaling remain to be determined, analysis of the cellular loci of function of this protein reveals a role for network activity in modulating pheromone responses in dauer formation.

### Tau tubulin kinases regulate pheromone-induced dauer formation

**Figure 5:**
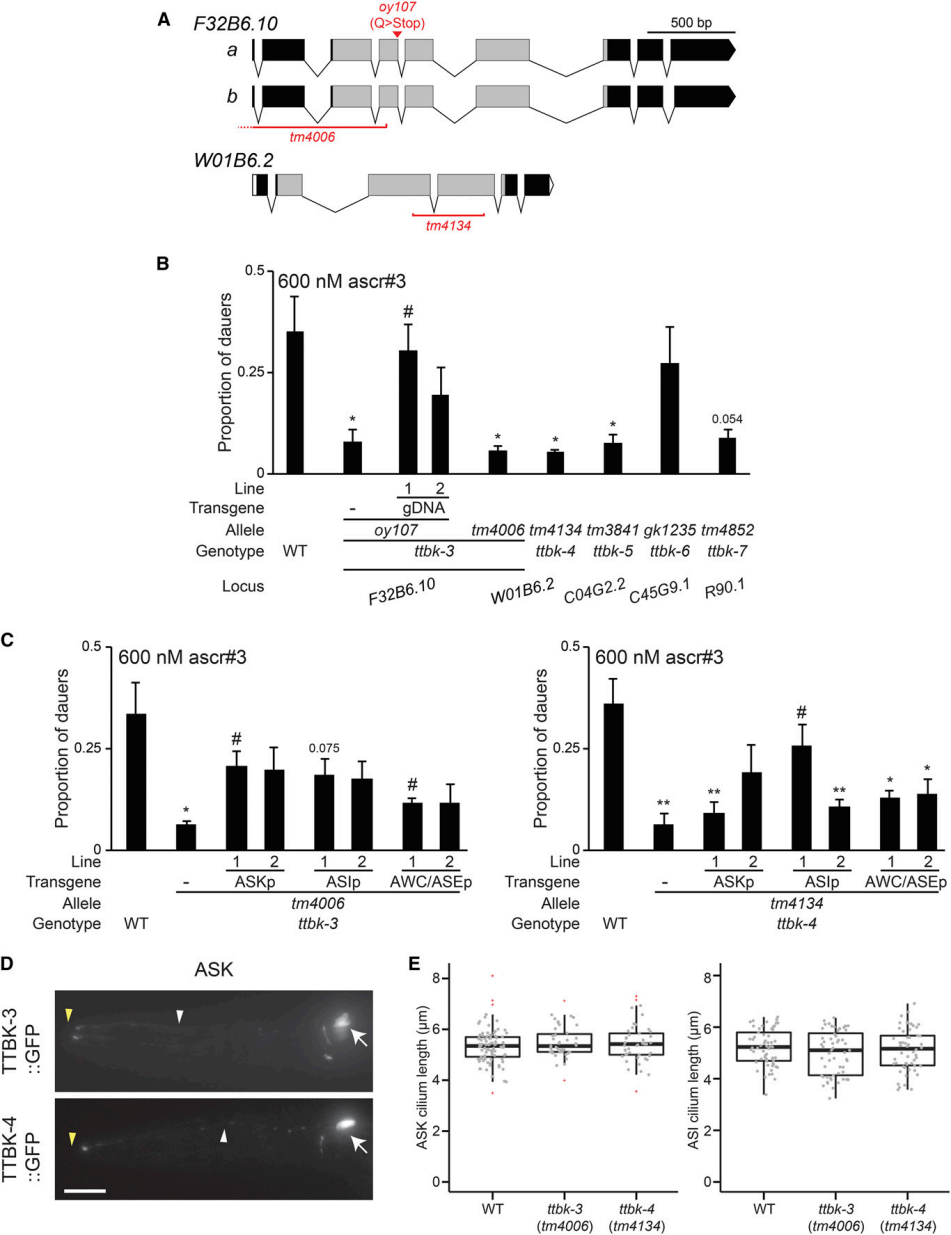
Multiple members of the TTBK (tau tubulin kinase-like) protein family regulate pheromone-induced dauer formation. (A) Predicted exon/intron structures of *F32B6.10 (ttbk-3)* and *W01B6.2 (ttbk-4)*. Gray boxes indicate exons predicted to encode the kinase domains. The location and nature of the mutations in *F32B6.10* and *W01B6.2* alleles are indicated. (B and C) Dauers formed by animals of the indicated genotypes in response to 600 nM ascr#3 at 25°. Lines refer to independent transgenic strains carrying the indicated transgenes on extrachromosomal arrays. *ttbk* cDNAs were expressed in ASK under the *sra-9* promoter, in ASI under the *srg-47* promoter, and in AWC/ASE under the *ceh-36* promoter. Each data point is the average of at least three biologically independent assays of 40–110 animals each. Errors are SEM.* and ** indicate different from wild-type at *P* < 0.05 and 0.01, respectively; # indicates different from the relevant *ttbk* mutant at *P* < 0.05, 0.01, and 0.001, respectively (ANOVA and Games-Howell posthoc test). (D) Representative images of the localization patterns of TTBK-3 and TTBK-4 fusion proteins in ASK. Arrows, white and yellow arrowheads indicate cell soma, dendrites and cilia, respectively. Anterior is at left. Scale: 10 μm. (E) Lengths of ASK (left) and ASI (right) cilia in animals of the indicated genotypes. Wild-type data are repeated from Figure 3C. Cilia were visualized via expression of *srbc-66*p::*mCherry* (ASK) and *str-3*p::*gfp* (ASI). Top and bottom bounds of boxes indicate 25th and 75th percentiles, respectively. Medians are indicated by thick horizontal bars. Outliers are indicated by red + symbols. ascr, ascaroside; gDNA, genomic DNA; GFP, green fluorescent protein; SEM, standard errors of the mean; WT, wild-type.

The *oy107* mutation is predicted to result in a truncated protein encoded by the *F32B6.10* gene (Figure 5A). The ascr#3-induced dauer formation defects of *oy107* mutants were partly rescued upon expression of wild-type *F32B6.10* genomic sequences (Figure 5B). In addition, animals carrying the *F32B6.10(tm4006)* deletion allele (Figure 5A) exhibited dauer formation defects similar to those exhibited by *oy107* mutants (Figure 5B), indicating that *oy107* is an allele of *F32B6.10*.

*F32B6.10* encodes a tau tubulin kinase (TTBK)-like enzyme belonging to the casein kinase I (CK1) superfamily of kinases (Manning 2005; Ikezu and Ikezu 2014). TTBK proteins were originally identified by their ability to phosphorylate the microtubule-associated protein tau, as well as tubulin (Takahashi *et al.* 1995; Tomizawa *et al.* 2001; Sato *et al.* 2006). The presence of hyper-phosphorylated tau in brain neurofibrillary tangles is a characteristic of tauopathies, including Alzheimer’s disease (Grundke-Iqbal *et al.* 1986; Wolozin *et al.* 1986; Iqbal *et al.* 1989), and genetic variants in *TTBK1* have been associated with the late-onset form of this disease (Vazquez-Higuera *et al.* 2011; Yu *et al.* 2011). In contrast to *TTBK1*, whose expression is restricted to the central nervous system (Sato *et al.* 2006; Lund *et al.* 2013), *TTBK2* is expressed more broadly in multiple tissues in mammals (Houlden *et al.* 2007). *TTBK2* has recently been shown to play a role in ciliogenesis and cellular processes such as regulation of transporter activity (Alesutan *et al.* 2012; Almilaji *et al.* 2013; Liao *et al.* 2015). Variants in *TTBK2* have been linked to spinocerebellar ataxia (Houlden *et al.* 2007), although the causal relationship between *TTBK2* function and this neurodegenerative disease is unclear. Interestingly, the TTBK family appears to be greatly expanded in *C. elegans*, with the *C. elegans* genome predicted to encode 32 TTBK-like proteins (Manning 2005). A subset of these kinases belonging to the larger superfamily has been analyzed in *C. elegans* models of neurodegenerative diseases (Kraemer *et al.* 2006; Liachko *et al.* 2014), but the endogenous functions of these proteins are largely unknown.

We noted that genes encoding two additional TTBK-like enzymes, *W01B6.2* and *C04G2.2*, are located within 200 kb of *F32B6.10* on linkage group IV (www.wormbase.org). Given the sequence homology, we asked whether mutations in one or both of these linked *ttbk* genes also affect dauer formation. As shown in Figure 5B, loss of the linked *W01B6.2* and *C04G2.2*, but not of the unlinked *C45G9.1* TTBK-like genes, resulted in dauer formation defects similar to those exhibited by *F32B6.10* mutants. Mutations in the unlinked *R90.1 ttbk* gene also resulted in pheromone-induced dauer formation defects (Figure 5B). These observations suggest that a subset of these kinases contributes to pheromone-induced dauer formation. We henceforth refer to *F32B6.10* as *ttbk-3*, *W01B6.2* as *ttbk-4*, *C04G2.2* as *ttbk-5*, *C45G9.1* as *ttbk-6*, and *R90.1* as *ttbk-7*.

We characterized the expression patterns of *ttbk-3-7* by examining the expression of *gfp* driven by their upstream regulatory sequences. Expression of transcriptional *ttbk*p::*gfp* fusion genes was weak and variable, but was observed primarily in neurons, including subsets of sensory neurons in the amphid sense organs of the head (Figure S2). In particular, *ttbk-3*, *ttbk-4*, and *ttbk-5* transcriptional fusion genes were expressed in either or both of the pheromone-sensing ASK and ASI sensory neurons (Figure S2). Additional expressing neurons could not be reliably identified due to variable and weak expression of these reporter genes. We next performed cell-specific rescue experiments to identify the site(s) of action of *ttbk-3* and *ttbk-4* in the regulation of dauer formation. Expression of *ttbk-3* and *ttbk-4* in either ASI or ASK rescued ascr#3-induced dauer formation defects of *ttbk-3(tm4006)* and *ttbk-4(tm4134)* mutants more strongly than expression in the AWC/ASE sensory neurons (Figure 5, C and D). Functional GFP-tagged TTBK-3 and TTBK-4 fusion proteins were localized throughout the cell in ASK, but appeared to be excluded from sensory cilia (Figure 5E). These results indicate that TTBK-3 and TTBK-4 function in ASI and ASK can regulate dauer formation.

Since TTBK proteins have been implicated in the regulation of neuronal morphology and ciliogenesis, we asked whether neuronal morphology, including ciliary morphology, is altered in these mutants. The pattern and extent of dye uptake by a subset of sensory neurons, including the ASK and ASI neurons, was unaffected in *ttbk-3(oy107)* mutants (Table 1), indicating that these neurons are generated and specified, and that their ciliary sensory endings are grossly unaffected in this mutant background. Dye-filling was also unaffected in *ttbk-4(tm4134)* mutants (100% adults filled with dye; *n* = 40). We also directly visualized ASI and ASK neuronal morphology via the expression of soluble *gfp* driven under cell-specific reporters. The positions of ASI and ASK cell soma and cellular and ciliary morphologies did not appear to be grossly altered in examined neurons in *ttbk-3* or *ttbk-4* mutants (Figure 5E and Figure S3). We conclude that TTBK-3 and TTBK-4 do not regulate sensory neuron cellular architecture to regulate pheromone-induced dauer formation.

### Conclusions

In summary, we have shown that we successfully identified mutations in genes required for dauer formation using pheromone-mediated *str-3*p::*gfp* repression as a screening tool. This screening strategy can now be readily scaled up and saturated via the use of automated sorting devices (Doitsidou *et al.* 2008; Crane *et al.* 2012; Entchev *et al.* 2015). Our pilot screen allowed us to identify genes such as *che-12*, mutations in which affect sensory neuron development and/or morphology. We also described new roles for previously identified genes such as *maco-1* and *qui-1* in the regulation of dauer formation. Moreover, analysis of the role of *qui-1* in dauer formation allowed us to demonstrate that pheromone-induced dauer formation is modulated as a function of environmental noxious chemicals, and that this modulation is mediated by QUI-1-dependent activity of the ASH and ADL nociceptive neurons. We also identified a subfamily of TTBK-like enzymes that plays a role in dauer formation, suggesting that further analyses of TTBK protein function in this process may provide new insights into the roles of these conserved molecules in the regulation of sensory neuron function. Continued investigation of mutants and genes isolated in this and related screens in the future will allow us to better understand how animals sense and integrate environmental information to drive critical binary developmental decisions.

## Supplementary Material

Supplemental Material
