# Supplementary material for: A Forward Genetic Screen for Molecules Involved in Pheromone-Induced Dauer Formation in Caenorhabditis elegans
Source: G3 (Bethesda). 2016 Mar 10;6(5):1475–87. doi: 10.1534/g3.115.026450 (PMC4856098; doi:10.1534/g3.115.026450)
Supplement: Supplemental Material [file supp_6_5_1475__index.html]

A Forward Genetic Screen for Molecules Involved in Pheromone-Induced Dauer Formation in Caenorhabditis elegans — Supplemental Material 

# A Forward Genetic Screen for Molecules Involved in Pheromone-Induced Dauer Formation in *Caenorhabditis elegans*

## Supplemental Material for Neal *et al.*, 2016

**Files in this Data Supplement:**

- Supplemental Material - File contains Table S1-S3, and also legends for Figures S1-S3. (.pdf, 332 KB)
- Table S1 - List of strains used in this work. (.pdf, 299 KB)
- Table S2 - Dauer formation defects of *phd* mutants. (.pdf, 132 KB)
- Table S3 - Whole genome resequencing metrics. (.pdf, 14 KB)
- Figure S1 - Expression of *daf-7*p::*gfp* is unaffected in *maco-1* mutants. (.ai, 1,920 KB)
- Figure S2 - Expression patterns of *ttbkp*::*gfp* fusion genes. (.ai, 2,007 KB)
- Figure S3  - ASK and ASI cellular and cilia morphologies are unaffected in *ttbk-3* and *ttbk-4* mutants. (.ai, 2,400 KB)
