# Supplementary material for: A Forward Genetic Screen for Molecules Involved in Pheromone-Induced Dauer Formation in Caenorhabditis elegans
Source: G3 (Bethesda). 2016 Mar 10;6(5):1475–87. doi: 10.1534/g3.115.026450 (PMC4856098; doi:10.1534/g3.115.026450)
Supplement: Supplemental Material [file supp_g3.115.026450_026450SM.pdf]

SUPPLEMENTAL MATERIAL

Supplemental Figure Legends

Figure S1. Expression of *daf-7*p*::gfp* is unaffected in *maco-1* mutants. Shown are representative images of *daf-7*p*::gfp* expression in ASI neurons (arrows) of wild-type and *maco-1(ok3165)* animals. In red are cells filled with DiI. Scale bar: 5 μm.

Figure S2. Expression patterns of *ttbk*p*::gfp* fusion genes. Shown are representative images of the heads of animals expressing the indicated transcriptional fusion genes. A schematic of sensory neurons filling with DiI (red) is shown at top. White and yellow arrows indicate ASI and ASK cell bodies, respectively. Expression of the coinjection marker in the process of the I5 neuron is indicated with a white arrowhead. Anterior is at left. Scale: 5 μm.

Figure S3. ASK and ASI cellular and cilia morphologies are unaffected in *ttbk-3* and *ttbk-4* mutants.

Representative images of ASK and ASI neurons visualized using cell-specific reporters. Soma, dendrites and cilia are indicated by white arrows, white arrowheads and yellow arrowheads, respectively. Anterior is at left. Scale bar: 10 μm.

Table S1. List of strains used in this work.

Strain	Genotype	Source and/or parent strains[a]	Relevant Tables and Figures
WT	N2 (Bristol)	CGC	Tables 1, S2; Figs 2, 3B, 3E, 3F, 4B, 4C, 4E, 5B, 5C
CX3596	*kyIs128(str-3*p*::gfp; lin-15+)*X	(Peckol *et al.* 2001)	Tables 1, S2; Figs 2, 3C, 5E, S3
PY6561	*srbc-64(tm1946)* I; *srbc-66(tm2943)* V	(Kim *et al.* 2009)	Tables 1, S2; Fisg 2, 4E
KHK742	*srbc-64(tm1946)* I; *srbc-66(tm2943)* V; *kyIs128(str-3*p*::gfp; lin-15+)* X	PY6561, CX3596	Table 1
KHK787	*srg-36 srg-37(kyIr88), kyIs128(str-3*p*::gfp; lin-15+)* X	PY8244, CX3596	Table 1
PY8300	*oy103; kyIs128(str-3*p*::gfp; lin-15+)* X	CX3596	Tables 1, S2; Fig 2
PY8301	*oy104; kyIs128(str-3*p*::gfp; lin-15+)* X	CX3596	Tables 1, S2; Fig 2
PY8302	*oy105; kyIs128(str-3*p*::gfp; lin-15+)* X	CX3596	Tables 1, S2; Figs 2, 4B, 4C
PY8303	*oy106; kyIs128(str-3*p*::gfp; lin-15+)* X	CX3596	Tables 1, S2; Figs 2, 3B, 3C
PY8304	*oy107; kyIs128(str-3*p*::gfp; lin-15+)* X	CX3596	Tables 1, S2; Figs 2, 5B
PY8373	*oy108; kyIs128(str-3*p*::gfp; lin-15+)* X	CX3596	Tables 1, S2; Figs 2, 3E, 3F
PY10757	*oy109; kyIs128(str-3*p*::gfp; lin-15+)* X	CX3596	Tables 1, S2; Fig 2
PY10754	*oy113; kyIs128(str-3*p*::gfp; lin-15+)* X	CX3596	Table 1
PY10759	*oy117; kyIs128(str-3*p*::gfp; lin-15+)* X	CX3596	Table 1
PY10760	*oy118; kyIs128(str-3*p*::gfp; lin-15+)* X	CX3596	Table 1
PY10761	*oy119; kyIs128(str-3*p*::gfp; lin-15+)* X	CX3596	Table 1
PY10762	*oy120; kyIs128(str-3*p*::gfp; lin-15+)* X	CX3596	Table 1
PY10767	*oy125; kyIs128(str-3*p*::gfp; lin-15+)* X	CX3596	Table 1
PY10768	*oy126; kyIs128(str-3*p*::gfp; lin-15+)* X	CX3596	Table 1
PY10769	*oy127; kyIs128(str-3*p*::gfp; lin-15+)* X	CX3596	Table 1

PY10771	*oy129; kyIs128(str-3*p*::gfp; lin-15+)* X	CX3596	Table 1
PY10773	*oy131; kyIs128(str-3*p*::gfp; lin-15+)* X	CX3596	Table 1
PY10776	*oy134; kyIs128(str-3*p*::gfp; lin-15+)* X	CX3596	Table 1
PY10777	*oy135; kyIs128(str-3*p*::gfp; lin-15+)* X	CX3596	Table 1
PY10779	*oy137; kyIs128(str-3*p*::gfp; lin-15+)* X	CX3596	Table 1
PY10780	*oy138; kyIs128(str-3*p*::gfp; lin-15+)* X	CX3596	Table 1
PY10781	*oy139; kyIs128(str-3*p*::gfp; lin-15+)* X	CX3596	Table 1
PY10782	*oy140; kyIs128(str-3*p*::gfp; lin-15+)* X	CX3596	Table 1
PY10783	*oy141; kyIs128(str-3*p*::gfp; lin-15+)* X	CX3596	Table 1
PY10784	*oy142; kyIs128(str-3*p*::gfp; lin-15+)* X	CX3596	Table 1
PY10785	*oy143; kyIs128(str-3*p*::gfp; lin-15+)* X	CX3596	Table 1
PY10786	*oy144; kyIs128(str-3*p*::gfp; lin-15+)* X	CX3596	Table 1
PY10792	*oy106; kyIs128(str-3*p*::gfp; lin-15+)* X; Ex[*che-12* gDNA, *unc-122*p::*gfp*] line 1	injected into PY8303	Fig 3B
PY10793	*oy106; kyIs128(str-3*p*::gfp; lin-15+)* X; Ex[*che-12* gDNA, *unc-122*p::*gfp*] line 2	injected into PY8303	Fig 3B
PY10869	*oy106; kyIs128(str-3*p*::gfp; lin-15+)* X; Ex[*che-12*p*::che-12::gfp, unc-122*p::*dsRed*] line 1	injected into PY8303	Fig 3B
PY10870	*oy106; kyIs128(str-3*p*::gfp; lin-15+)* X; Ex[*che-12*p*::che-12::gfp, unc-122*p::*dsRed*] line 2	injected into PY8303	Fig 3B
CB3332	*che-12(e1812)* V	CGC	Fig 3B
PY10833	*che-12(e1812)* V*;* Ex[*che-12* gDNA, *unc-122*p*::gfp*] line 1	injected into CB3332	Fig 3B
PY10834	*che-12(e1812)* V*;* Ex[*che-12* gDNA, *unc-122*p*::gfp*] line 2	injected into CB3332	Fig 3B
PY10871	*Ex[srbc-66*p*::mCherry, unc-122*p*::dsRed]*	injected into WT	Figs 3C, 5E, S3
PY10872	*oy106; Ex[srbc-66*p*::mCherry,*	injected into	Fig 3C

	*unc-122*p*::dsRed]*	PY8303	
PY10873	*che-12(e1812)* V*; Ex[srbc-66*p*::mCherry, unc-122*p*::dsRed]*	injected into CB3332	Fig 3C
RB2329	*maco-1*(*ok3165*) I	CGC	Figs 3E, 3F
PY10796	*oy108*; *kyIs128(str-3*p*::gfp; lin-15+)* X; Ex[*maco-1* gDNA, *unc-122*p*::gfp*] line 1	injected into PY8373	Figs 3E, 3F
PY10797	*oy108*; *kyIs128(str-3*p*::gfp; lin-15+)* X; Ex[*maco-1* gDNA, *unc-122*p*::gfp*] line 2	injected into PY8373	Figs 3E, 3F
KHK728	*oy108*; *kyIs128(str-3*p*::gfp; lin-15+)* X; Ex[*srg-47*p::*maco-1*, *unc-122*p*::gfp*] line 1	injected into PY8373	Figs 3E, 3F
KHK729	*oy108*; *kyIs128(str-3*p*::gfp; lin-15+)* X; Ex[*srg-47*p::*maco-1*, *unc-122*p*::gfp*] line 2	injected into PY8373	Figs 3E, 3F
KHK733	*oy108*; *kyIs128(str-3*p*::gfp; lin-15+)* X; Ex[*sra-9*p::*maco-1*, *unc-122*p*::gfp*] line 1	injected into PY8373	Figs 3E, 3F
KHK734	*oy108*; *kyIs128(str-3*p*::gfp; lin-15+)* X; Ex[*sra-9*p::*maco-1*, *unc-122*p*::gfp*] line 2	injected into PY8373	Figs 3E, 3F
KHK739	*oy108*; *kyIs128(str-3*p*::gfp; lin-15+)* X; Ex[*trx-1*p::*maco-1*, *unc-122*p*::gfp*] line 1	injected into PY8373	Figs 3E, 3F
KHK740	*oy108*; *kyIs128(str-3*p*::gfp; lin-15+)* X; Ex[*trx-1*p::*maco-1*, *unc-122*p*::gfp*] line 2	injected into PY8373	Figs 3E, 3F
PY10798	*qui-1*(*gb404*) IV	outcrossed from NA404	Figs 4B, 4C, 4E
PY10790	*oy105; kyIs128(str-3*p*::gfp; lin-15+)* X; Ex[*qui-1* gDNA, *unc-122*p::*gfp*] line 1	injected into PY8302	Figs 4B, 4C
PY10791	*oy105; kyIs128(str-3*p*::gfp; lin-15+)* X; Ex[*qui-1* gDNA, *unc-122*p::*gfp*] line 2	injected into PY8302	Figs 4B, 4C
PY8377	*oy105; kyIs128(str-3*p*::gfp; lin-15+)* X; Ex[*sra-6*p*::qui-1*, *unc-122*p::*gfp*] line 1	injected into PY8302	Figs 4B, 4C
PY8378	*oy105; kyIs128(str-3*p*::gfp; lin-15+)* X; Ex[*sre-1Δ*p*::qui-1*, *unc-122*p::*gfp*] line 1	injected into PY8302	Figs 4B, 4C
PY8379	*oy105; kyIs128(str-3*p*::gfp; lin-15+)* X; Ex[*sra-6*p*::qui-1*, *sre-1Δ*p*::qui-1*, *unc-122*p::*gfp*]	injected into PY8302	Figs 4B, 4C

	line 1		
PY8376	*oy105; kyIs128(str-3*p*::gfp; lin-15+)* X; Ex[*sra-9*p*::qui-1*, *unc-122*p::*gfp*] line 1	injected into PY8302	Figs 4B, 4C
KHK741	Ex[*sra-6*p*::qui-1::mCherry*, *rol-6(su1006)*]	injected into WT	Fig 4D
PY10794	*oy107; kyIs128(str-3*p*::gfp; lin-15+)* X; Ex[*F32B6.10* gDNA, *unc-122*p*::gfp*] line 1	injected into PY8304	Fig 5B
PY10795	*oy107; kyIs128(str-3*p*::gfp; lin-15+)* X; Ex[*F32B6.10* gDNA, *unc-122*p*::gfp*] line 2	injected into PY8304	Fig 5B
PY10799	*ttbk-3/F32B6.10*(*tm4006*) IV	NBRP	Figs 5B, 5C
PY10800	*ttbk-4/W01B6.2*(*tm4134*) IV	NBRP	Figs 5B, 5C
PY10801	*ttbk-5/C04G2.2*(*tm3841*) IV	NBRP	Fig 5B
VC2697	*ttbk-6/C45G9.1*(*gk1235*) III	CGC	Fig 5B
PY10802	*ttbk-7/R90.1*(*tm4852*) V	NBRP	Fig 5B
PY10874	*ttbk-3(tm4006)* IV; Ex[*sra-9*p::*ttbk-3::gfp* (1 ng/μl), *unc-122*p::*dsRed*] line 1	injected into PY10799	Fig 5C
PY10875	*ttbk-3(tm4006)* IV; Ex[*sra-9*p::*ttbk-3::gfp* (1 ng/μl), *unc-122*p::*dsRed*] line 2	injected into PY10799	Fig 5C
PY10876	*ttbk-3(tm4006)* IV; Ex[*srg-47*p::*ttbk-3::gfp* (1 ng/μl), *unc-122*p::*dsRed*] line 1	injected into PY10799	Fig 5C
PY10877	*ttbk-3(tm4006)* IV; Ex[*srg-47*p::*ttbk-3::gfp* (1 ng/μl), *unc-122*p::*dsRed*] line 2	injected into PY10799	Fig 5C
PY10878	*ttbk-3(tm4006)* IV; Ex[*ceh-36*p::*ttbk-3::gfp* (1 ng/μl), *unc-122*p::*dsRed*] line 1	injected into PY10799	Fig 5C
PY10879	*ttbk-3(tm4006)* IV; Ex[*ceh-36*p::*ttbk-3::gfp* (1 ng/μl), *unc-122*p::*dsRed*] line 2	injected into PY10799	Fig 5C
PY10880	*ttbk-4(tm4134)* IV; Ex[*sra-9*p::*ttbk-4::gfp* (1 ng/μl), *unc-122*p::*dsRed*] line 1	injected into PY10800	Fig 5C
PY10881	*ttbk-4(tm4134)* IV; Ex[*sra-9*p::*ttbk-4::gfp* (1 ng/μl), *unc-122*p::*dsRed*] line 2	injected into PY10800	Fig 5C
PY10882	*ttbk-4(tm4134)* IV; Ex[*srg-47*p::*ttbk-4::gfp* (1 ng/μl), *unc-122*p::*dsRed*] line 1	injected into PY10800	Fig 5C
PY10883	*ttbk-4(tm4134)* IV; Ex[*srg-47*p::*ttbk-4::gfp* (1 ng/μl), *unc-*	injected into PY10800	Fig 5C

	*122*p::*dsRed*] line 2		
PY10884	*ttbk-4(tm4134)* IV; Ex[*ceh-36*p::*ttbk-4::gfp* (1 ng/µl), *unc-122*p::*dsRed*] line 1	injected into PY10800	Fig 5C
PY10885	*ttbk-4(tm4134)* IV; Ex[*ceh-36*p::*ttbk-4::gfp* (1 ng/µl), *unc-122*p::*dsRed*] line 2	injected into PY10800	Fig 5C
PY10886	Ex[*sra-9*p::*ttbk-3::gfp* (15 ng/µl), *unc-122*p::*gfp*]	injected into WT	Fig 5D
PY10888	Ex[*sra-9*p::*ttbk-4::gfp* (15 ng/µl), *unc-122*p::*gfp*]	injected into WT	Fig 5D
PY10890	*ttbk-3(tm4006)* IV; Ex[*srbc-66*p*::mCherry, unc-122*p*::dsRed*]	injected into PY10799	Figs 5E, S3
PY10891	*ttbk-4(tm4134)* IV; Ex[*srbc-66*p*::mCherry, unc-122*p*::dsRed*]	injected into PY10800	Figs 5E, S3
PY10892	*ttbk-3(tm4006)* IV; *kyIs128(str-3*p*::gfp; lin-15+)* X	CX3596, PY10799	Figs 5E, S3
PY10893	*ttbk-4(tm4134)* IV; *kyIs128(str-3*p*::gfp; lin-15+)* X	CX3596, PY10800	Figs 5E, S3
FK181	*ksIs2*[*daf-7*p*::gfp, rol-6(su1006)*]	CGC	Fig S1
KHK743	*maco-1*(*ok3165*) I; *ksIs2*[*daf-7*p*::gfp, rol-6(su1006)*]	FK181, RB2329	Fig S1
PY10894	Ex[*ttbk-3*p*::gfp, unc-122*p*::dsRed*]	injected into WT	Fig S2
PY10895	Ex[*ttbk-4*p*::gfp, unc-122*p*::dsRed*]	injected into WT	Fig S2
PY10896	Ex[*ttbk-5*p*::gfp, unc-122*p*::dsRed*]	injected into WT	Fig S2
PY10897	Ex[*ttbk-6*p*::gfp, unc-122*p*::dsRed*]	injected into WT	Fig S2
PY10898	Ex[*ttbk-7*p*::gfp, unc-122*p*::dsRed*]	injected into WT	Fig S2

[a]CGC – *Caenorhabditis* Genetics Center; NBRP – National BioResource Project

Table S2. Dauer formation defects of *phd* mutants.

	EtOH[c]	Ascaroside[b]														
		ascr#2 (nM)			ascr#3 (nM)			ascr#5 (nM)			ascr#8 (nM)			icas#9 (nM)		
Strain[a]	0	60	600	6000	60	600	6000	60	600	6000	6	60	600	6	60	600
WT	0.03± 0.01	0.38± 0.05	0.61± 0.06	0.79± 0.03	0.48± 0.05	0.39± 0.04	0.82± 0.05	0.10± 0.06	0.83± 0.04	0.93± 0.03	0.28± 0.08	0.75± 0.07	0.87± 0.02	0.29± 0.03	0.66± 0.05	0.25± 0.02
kyIs128	0.02± 0.00	0.41± 0.08	0.64± 0.06	0.82± 0.01	0.48± 0.09	0.74± 0.06	0.94± 0.01	0.16± 0.03	0.88± 0.03	0.91± 0.04	0.39± 0.08	0.62± 0.09	0.85± 0.05	0.40± 0.12	0.52± 0.09	0.55± 0.20
srbc-64; srbc-66	0.00± 0.00	0.09± 0.04	0.21± 0.08	0.33± 0.11	0.03± 0.01	0.05± 0.02	0.34± 0.15	0.01± 0.01	0.29± 0.15	0.62± 0.16	0.10± 0.06	0.30± 0.09	0.51± 0.09	0.01± 0.01	0.06± 0.04	0.07± 0.05
oy103	0.00± 0.00	0.03± 0.01	0.12± 0.03	0.28± 0.04	0.00± 0.00	0.01± 0.00	0.12± 0.05	0.02± 0.01	0.23± 0.13	0.79± 0.09	0.02± 0.01	0.11± 0.07	0.24± 0.06	0.03± 0.01	0.05± 0.01	0.02± 0.00
oy104	0.00± 0.00	0.12± 0.05	0.36± 0.07	0.29± 0.04	0.00± 0.00	0.00± 0.00	0.05± 0.02	0.00± 0.00	0.39± 0.20	0.65± 0.18	0.05± 0.04	0.38± 0.14	0.66± 0.09	0.01± 0.01	0.03± 0.02	0.01± 0.00
oy105	0.08± 0.03	0.22± 0.09	0.18± 0.10	0.25± 0.10	0.10± 0.02	0.15± 0.06	0.29± 0.16	0.05± 0.03	0.31± 0.17	0.39± 0.16	0.33± 0.17	0.44± 0.12	0.58± 0.15	0.09± 0.03	0.38± 0.13	0.24± 0.11
oy106	0.02± 0.02	0.02± 0.01	0.04± 0.01	0.20± 0.08	0.10± 0.09	0.13± 0.10	0.31± 0.19	0.03± 0.02	0.42± 0.07	0.80± 0.04	0.02± 0.01	0.17± 0.08	0.54± 0.18	0.03± 0.01	0.14± 0.06	0.39± 0.14
oy107	0.00± 0.00	0.13± 0.06	0.23± 0.06	0.34± 0.12	0.02± 0.02	0.16± 0.06	0.41± 0.09	0.02± 0.01	0.29± 0.15	0.70± 0.13	0.19± 0.06	0.41± 0.06	0.60± 0.08	0.06± 0.02	0.17± 0.02	0.10± 0.03
oy108	0.03± 0.01	0.15± 0.07	0.10± 0.04	0.29± 0.11	0.04± 0.02	0.07± 0.06	0.33± 0.22	0.13± 0.09	0.20± 0.09	0.54± 0.15	0.02± 0.01	0.35± 0.17	0.38± 0.16	0.00± 0.00	0.11± 0.08	0.03± 0.02
oy109	0.04± 0.03	0.02± 0.02	0.02± 0.01	0.04± 0.02	0.02± 0.01	0.02± 0.00	0.04± 0.01	0.06± 0.04	0.03± 0.01	0.07± 0.03	0.02± 0.01	0.03± 0.01	0.10± 0.04	0.02± 0.01	0.03± 0.00	0.01± 0.00

[a]All strains, except WT and *srbc-64*; *srbc-66,* contain stably integrated copies of the *str-3*p*::gfp* fusion gene (*kyIs128*).
[b]Shown are the mean±SEM proportion of dauers formed for the given strains and conditions at 25°C. Data are from at least 3 biologically independent trials of 40-110 animals per assay, with at least 2 technical replicates in each trial.
[c]Ethanol was used as the diluent for all tested ascarosides compounds.

Table S3. Whole genome resequencing metrics.

Metric	Strain						
	oy103	*oy104*	*oy105*	*oy106*	*oy107*	*oy108*	*oy109*
Average genome coverage[a]	29.7X	33.2X	23.1X	23.3X	32.6X	33.8X	36.8X
Variants[b]	3659	3783	3783	3767	3735	3670	4441
Genic variants[c]	317	345	341	312	370	244	356
Unique coding variants[d]	102	118	129	97	148	31	132
Unique nonsense mutations[e]	4	3	8	3	3	0	3
Unique uncovered regions[f]	1	2	13	12	14	18	230

[a]Average depth of read coverage across unique regions in the genome.

[b]Total number of detected variants with respect to the N2 reference genome, including genic and intergenic regions. Only those variants that are supported by at least five overlapping consensus reads are reported in all cases.

[c]Variants in genic regions.

[d]Unique variants in coding regions present in individual genomes.

[e]Unique variants predicted to encode a premature termination codon in at least one isoform of a given locus in individual genomes.

[f]Genic regions lacking coverage in a given strain, but which are covered by reads in other sequenced strains.

REFERENCES

Kim, K., K. Sato, M. Shibuya, D. M. Zeiger, R. A. Butcher *et al.*, 2009 Two chemoreceptors mediate developmental effects of dauer pheromone in *C. elegans*. Science **326:** 994-998.

Peckol, E. L., E. R. Troemel and C. I. Bargmann, 2001 Sensory experience and sensory activity regulate chemosensory receptor gene expression in *C. elegans*. Proc. Natl. Acad. Sci. USA **98:** 11032-11038.
