# Supplementary material for: A Forward Genetic Screen for Molecules Involved in Pheromone-Induced Dauer Formation in Caenorhabditis elegans
Source: G3 (Bethesda). 2016 Mar 10;6(5):1475–87. doi: 10.1534/g3.115.026450 (PMC4856098; doi:10.1534/g3.115.026450)
Supplement: Supplemental Material [file supp_g3.115.026450_TableS1.pdf]

Table S1. List of strains used in this work.

Strain	Genotype	Source and/or parent strains[a]	Relevant Tables and Figures
WT	N2 (Bristol)	CGC	Tables 1, S2; Figs 2, 3B, 3E, 3F, 4B, 4C, 4E, 5B, 5C
CX3596	*kyIs128(str-3*p*::gfp; lin-15+)*X	(Peckol *et al.* 2001)	Tables 1, S2; Figs 2, 3C, 5E, S3
PY6561	*srbc-64(tm1946)* I; *srbc-66(tm2943)* V	(Kim *et al.* 2009)	Tables 1, S2; Fisg 2, 4E
KHK742	*srbc-64(tm1946)* I; *srbc-66(tm2943)* V; *kyIs128(str-3*p*::gfp; lin-15+)* X	PY6561, CX3596	Table 1
KHK787	*srg-36 srg-37(kyIr88), kyIs128(str-3*p*::gfp; lin-15+)* X	PY8244, CX3596	Table 1
PY8300	*oy103; kyIs128(str-3*p*::gfp; lin-15+)* X	CX3596	Tables 1, S2; Fig 2
PY8301	*oy104; kyIs128(str-3*p*::gfp; lin-15+)* X	CX3596	Tables 1, S2; Fig 2
PY8302	*oy105; kyIs128(str-3*p*::gfp; lin-15+)* X	CX3596	Tables 1, S2; Figs 2, 4B, 4C
PY8303	*oy106; kyIs128(str-3*p*::gfp; lin-15+)* X	CX3596	Tables 1, S2; Figs 2, 3B, 3C
PY8304	*oy107; kyIs128(str-3*p*::gfp; lin-15+)* X	CX3596	Tables 1, S2; Figs 2, 5B
PY8373	*oy108; kyIs128(str-3*p*::gfp; lin-15+)* X	CX3596	Tables 1, S2; Figs 2, 3E, 3F
PY10757	*oy109; kyIs128(str-3*p*::gfp; lin-15+)* X	CX3596	Tables 1, S2; Fig 2
PY10754	*oy113; kyIs128(str-3*p*::gfp; lin-15+)* X	CX3596	Table 1
PY10759	*oy117; kyIs128(str-3*p*::gfp; lin-15+)* X	CX3596	Table 1
PY10760	*oy118; kyIs128(str-3*p*::gfp; lin-15+)* X	CX3596	Table 1
PY10761	*oy119; kyIs128(str-3*p*::gfp; lin-15+)* X	CX3596	Table 1
PY10762	*oy120; kyIs128(str-3*p*::gfp; lin-15+)* X	CX3596	Table 1
PY10767	*oy125; kyIs128(str-3*p*::gfp; lin-15+)* X	CX3596	Table 1
PY10768	*oy126; kyIs128(str-3*p*::gfp; lin-15+)* X	CX3596	Table 1
PY10769	*oy127; kyIs128(str-3*p*::gfp; lin-15+)* X	CX3596	Table 1

PY10771	*oy129; kyIs128(str-3*p*::gfp; lin-15+)* X	CX3596	Table 1
PY10773	*oy131; kyIs128(str-3*p*::gfp; lin-15+)* X	CX3596	Table 1
PY10776	*oy134; kyIs128(str-3*p*::gfp; lin-15+)* X	CX3596	Table 1
PY10777	*oy135; kyIs128(str-3*p*::gfp; lin-15+)* X	CX3596	Table 1
PY10779	*oy137; kyIs128(str-3*p*::gfp; lin-15+)* X	CX3596	Table 1
PY10780	*oy138; kyIs128(str-3*p*::gfp; lin-15+)* X	CX3596	Table 1
PY10781	*oy139; kyIs128(str-3*p*::gfp; lin-15+)* X	CX3596	Table 1
PY10782	*oy140; kyIs128(str-3*p*::gfp; lin-15+)* X	CX3596	Table 1
PY10783	*oy141; kyIs128(str-3*p*::gfp; lin-15+)* X	CX3596	Table 1
PY10784	*oy142; kyIs128(str-3*p*::gfp; lin-15+)* X	CX3596	Table 1
PY10785	*oy143; kyIs128(str-3*p*::gfp; lin-15+)* X	CX3596	Table 1
PY10786	*oy144; kyIs128(str-3*p*::gfp; lin-15+)* X	CX3596	Table 1
PY10792	*oy106; kyIs128(str-3*p*::gfp; lin-15+)* X; Ex[*che-12* gDNA, *unc-122*p::*gfp*] line 1	injected into PY8303	Fig 3B
PY10793	*oy106; kyIs128(str-3*p*::gfp; lin-15+)* X; Ex[*che-12* gDNA, *unc-122*p::*gfp*] line 2	injected into PY8303	Fig 3B
PY10869	*oy106; kyIs128(str-3*p*::gfp; lin-15+)* X; Ex[*che-12*p::*che-12::gfp*, *unc-122*p::*dsRed*] line 1	injected into PY8303	Fig 3B
PY10870	*oy106; kyIs128(str-3*p*::gfp; lin-15+)* X; Ex[*che-12*p::*che-12::gfp*, *unc-122*p::*dsRed*] line 2	injected into PY8303	Fig 3B
CB3332	*che-12(e1812)* V	CGC	Fig 3B
PY10833	*che-12(e1812)* V*;* Ex[*che-12* gDNA, *unc-122*p*::gfp*] line 1	injected into CB3332	Fig 3B
PY10834	*che-12(e1812)* V*;* Ex[*che-12* gDNA, *unc-122*p*::gfp*] line 2	injected into CB3332	Fig 3B
PY10871	*Ex[srbc-66*p*::mCherry, unc-122*p*::dsRed]*	injected into WT	Figs 3C, 5E, S3
PY10872	*oy106; Ex[srbc-66*p*::mCherry,*	injected into	Fig 3C

	*unc-122*p*::dsRed]*	PY8303	
PY10873	*che-12(e1812)* V*; Ex[srbc-66*p*::mCherry, unc-122*p*::dsRed]*	injected into CB3332	Fig 3C
RB2329	*maco-1*(*ok3165*) I	CGC	Figs 3E, 3F
PY10796	*oy108*; *kyIs128(str-3*p*::gfp; lin-15+)* X; Ex[*maco-1* gDNA, *unc-122*p*::gfp*] line 1	injected into PY8373	Figs 3E, 3F
PY10797	*oy108*; *kyIs128(str-3*p*::gfp; lin-15+)* X; Ex[*maco-1* gDNA, *unc-122*p*::gfp*] line 2	injected into PY8373	Figs 3E, 3F
KHK728	*oy108*; *kyIs128(str-3*p*::gfp; lin-15+)* X; Ex[*srg-47*p::*maco-1*, *unc-122*p*::gfp*] line 1	injected into PY8373	Figs 3E, 3F
KHK729	*oy108*; *kyIs128(str-3*p*::gfp; lin-15+)* X; Ex[*srg-47*p::*maco-1*, *unc-122*p*::gfp*] line 2	injected into PY8373	Figs 3E, 3F
KHK733	*oy108*; *kyIs128(str-3*p*::gfp; lin-15+)* X; Ex[*sra-9*p::*maco-1*, *unc-122*p*::gfp*] line 1	injected into PY8373	Figs 3E, 3F
KHK734	*oy108*; *kyIs128(str-3*p*::gfp; lin-15+)* X; Ex[*sra-9*p::*maco-1*, *unc-122*p*::gfp*] line 2	injected into PY8373	Figs 3E, 3F
KHK739	*oy108*; *kyIs128(str-3*p*::gfp; lin-15+)* X; Ex[*trx-1*p::*maco-1*, *unc-122*p*::gfp*] line 1	injected into PY8373	Figs 3E, 3F
KHK740	*oy108*; *kyIs128(str-3*p*::gfp; lin-15+)* X; Ex[*trx-1*p::*maco-1*, *unc-122*p*::gfp*] line 2	injected into PY8373	Figs 3E, 3F
PY10798	*qui-1*(*gb404*) IV	outcrossed from NA404	Figs 4B, 4C, 4E
PY10790	*oy105; kyIs128(str-3*p*::gfp; lin-15+)* X; Ex[*qui-1* gDNA, *unc-122*p::*gfp*] line 1	injected into PY8302	Figs 4B, 4C
PY10791	*oy105; kyIs128(str-3*p*::gfp; lin-15+)* X; Ex[*qui-1* gDNA, *unc-122*p::*gfp*] line 2	injected into PY8302	Figs 4B, 4C
PY8377	*oy105; kyIs128(str-3*p*::gfp; lin-15+)* X; Ex[*sra-6*p*::qui-1*, *unc-122*p::*gfp*] line 1	injected into PY8302	Figs 4B, 4C
PY8378	*oy105; kyIs128(str-3*p*::gfp; lin-15+)* X; Ex[*sre-1Δ*p*::qui-1*, *unc-122*p::*gfp*] line 1	injected into PY8302	Figs 4B, 4C
PY8379	*oy105; kyIs128(str-3*p*::gfp; lin-15+)* X; Ex[*sra-6*p*::qui-1*, *sre-1Δ*p*::qui-1*, *unc-122*p::*gfp*]	injected into PY8302	Figs 4B, 4C

	line 1		
PY8376	*oy105; kyIs128(str-3*p*::gfp; lin-15+)* X; Ex[*sra-9*p*::qui-1*, *unc-122*p::*gfp*] line 1	injected into PY8302	Figs 4B, 4C
KHK741	Ex[*sra-6*p*::qui-1::mCherry*, *rol-6(su1006)*]	injected into WT	Fig 4D
PY10794	*oy107; kyIs128(str-3*p*::gfp; lin-15+)* X; Ex[*F32B6.10* gDNA, *unc-122*p*::gfp*] line 1	injected into PY8304	Fig 5B
PY10795	*oy107; kyIs128(str-3*p*::gfp; lin-15+)* X; Ex[*F32B6.10* gDNA, *unc-122*p*::gfp*] line 2	injected into PY8304	Fig 5B
PY10799	*ttbk-3/F32B6.10*(*tm4006*) IV	NBRP	Figs 5B, 5C
PY10800	*ttbk-4/W01B6.2*(*tm4134*) IV	NBRP	Figs 5B, 5C
PY10801	*ttbk-5/C04G2.2*(*tm3841*) IV	NBRP	Fig 5B
VC2697	*ttbk-6/C45G9.1*(*gk1235*) III	CGC	Fig 5B
PY10802	*ttbk-7/R90.1*(*tm4852*) V	NBRP	Fig 5B
PY10874	*ttbk-3(tm4006)* IV; Ex[*sra-9*p::*ttbk-3::gfp* (1 ng/µl), *unc-122*p::*dsRed*] line 1	injected into PY10799	Fig 5C
PY10875	*ttbk-3(tm4006)* IV; Ex[*sra-9*p::*ttbk-3::gfp* (1 ng/µl), *unc-122*p::*dsRed*] line 2	injected into PY10799	Fig 5C
PY10876	*ttbk-3(tm4006)* IV; Ex[*srg-47*p::*ttbk-3::gfp* (1 ng/µl), *unc-122*p::*dsRed*] line 1	injected into PY10799	Fig 5C
PY10877	*ttbk-3(tm4006)* IV; Ex[*srg-47*p::*ttbk-3::gfp* (1 ng/µl), *unc-122*p::*dsRed*] line 2	injected into PY10799	Fig 5C
PY10878	*ttbk-3(tm4006)* IV; Ex[*ceh-36*p::*ttbk-3::gfp* (1 ng/µl), *unc-122*p::*dsRed*] line 1	injected into PY10799	Fig 5C
PY10879	*ttbk-3(tm4006)* IV; Ex[*ceh-36*p::*ttbk-3::gfp* (1 ng/µl), *unc-122*p::*dsRed*] line 2	injected into PY10799	Fig 5C
PY10880	*ttbk-4(tm4134)* IV; Ex[*sra-9*p::*ttbk-4::gfp* (1 ng/µl), *unc-122*p::*dsRed*] line 1	injected into PY10800	Fig 5C
PY10881	*ttbk-4(tm4134)* IV; Ex[*sra-9*p::*ttbk-4::gfp* (1 ng/µl), *unc-122*p::*dsRed*] line 2	injected into PY10800	Fig 5C
PY10882	*ttbk-4(tm4134)* IV; Ex[*srg-47*p::*ttbk-4::gfp* (1 ng/µl), *unc-122*p::*dsRed*] line 1	injected into PY10800	Fig 5C
PY10883	*ttbk-4(tm4134)* IV; Ex[*srg-47*p::*ttbk-4::gfp* (1 ng/µl), *unc-*	injected into PY10800	Fig 5C

	*122*p::*dsRed*] line 2		
PY10884	*ttbk-4(tm4134)* IV; Ex[*ceh-36*p::*ttbk-4::gfp* (1 ng/µl), *unc-122*p::*dsRed*] line 1	injected into PY10800	Fig 5C
PY10885	*ttbk-4(tm4134)* IV; Ex[*ceh-36*p::*ttbk-4::gfp* (1 ng/µl), *unc-122*p::*dsRed*] line 2	injected into PY10800	Fig 5C
PY10886	Ex[*sra-9*p::*ttbk-3::gfp* (15 ng/µl), *unc-122*p::*gfp*]	injected into WT	Fig 5D
PY10888	Ex[*sra-9*p::*ttbk-4::gfp* (15 ng/µl), *unc-122*p::*gfp*]	injected into WT	Fig 5D
PY10890	*ttbk-3(tm4006)* IV; Ex[*srbc-66*p*::mCherry, unc-122*p*::dsRed*]	injected into PY10799	Figs 5E, S3
PY10891	*ttbk-4(tm4134)* IV; Ex[*srbc-66*p*::mCherry, unc-122*p*::dsRed*]	injected into PY10800	Figs 5E, S3
PY10892	*ttbk-3(tm4006)* IV; *kyIs128(str-3*p*::gfp; lin-15+)* X	CX3596, PY10799	Figs 5E, S3
PY10893	*ttbk-4(tm4134)* IV; *kyIs128(str-3*p*::gfp; lin-15+)* X	CX3596, PY10800	Figs 5E, S3
FK181	*ksIs2*[*daf-7*p*::gfp, rol-6(su1006)*]	CGC	Fig S1
KHK743	*maco-1*(*ok3165*) I; *ksIs2*[*daf-7*p*::gfp, rol-6(su1006)*]	FK181, RB2329	Fig S1
PY10894	Ex[*ttbk-3*p*::gfp, unc-122*p*::dsRed*]	injected into WT	Fig S2
PY10895	Ex[*ttbk-4*p*::gfp, unc-122*p*::dsRed*]	injected into WT	Fig S2
PY10896	Ex[*ttbk-5*p*::gfp, unc-122*p*::dsRed*]	injected into WT	Fig S2
PY10897	Ex[*ttbk-6*p*::gfp, unc-122*p*::dsRed*]	injected into WT	Fig S2
PY10898	Ex[*ttbk-7*p*::gfp, unc-122*p*::dsRed*]	injected into WT	Fig S2

[a]CGC – *Caenorhabditis* Genetics Center; NBRP – National BioResource Project

REFERENCES

Kim, K., K. Sato, M. Shibuya, D. M. Zeiger, R. A. Butcher *et al.*, 2009 Two chemoreceptors mediate developmental effects of dauer pheromone in *C. elegans*. Science **326:** 994-998.

Peckol, E. L., E. R. Troemel and C. I. Bargmann, 2001 Sensory experience and sensory activity regulate chemosensory receptor gene expression in *C. elegans*. Proc. Natl. Acad. Sci. USA **98:** 11032-11038.
