# Supplementary material for: A Forward Genetic Screen for Molecules Involved in Pheromone-Induced Dauer Formation in Caenorhabditis elegans
Source: G3 (Bethesda). 2016 Mar 10;6(5):1475–87. doi: 10.1534/g3.115.026450 (PMC4856098; doi:10.1534/g3.115.026450)
Supplement: Supplemental Material [file supp_g3.115.026450_TableS2.pdf]

Table S2. Dauer formation defects of *phd* mutants.

	EtOH[c]	Ascaroside[b]														
		ascr#2 (nM)			ascr#3 (nM)			ascr#5 (nM)			ascr#8 (nM)			icas#9 (nM)		
Strain[a]	0	60	600	6000	60	600	6000	60	600	6000	6	60	600	6	60	600
WT	0.03±0.01	0.38±0.05	0.61±0.06	0.79±0.03	0.48±0.05	0.39±0.04	0.82±0.05	0.10±0.06	0.83±0.04	0.93±0.03	0.28±0.08	0.75±0.07	0.87±0.02	0.29±0.03	0.66±0.05	0.25±0.02
kyIs128	0.02±0.00	0.41±0.08	0.64±0.06	0.82±0.01	0.48±0.09	0.74±0.06	0.94±0.01	0.16±0.03	0.88±0.03	0.91±0.04	0.39±0.08	0.62±0.09	0.85±0.05	0.40±0.12	0.52±0.09	0.55±0.20
srbc-64; srbc-66	0.00±0.00	0.09±0.04	0.21±0.08	0.33±0.11	0.03±0.01	0.05±0.02	0.34±0.15	0.01±0.01	0.29±0.15	0.62±0.16	0.10±0.06	0.30±0.09	0.51±0.09	0.01±0.01	0.06±0.04	0.07±0.05
oy103	0.00±0.00	0.03±0.01	0.12±0.03	0.28±0.04	0.00±0.00	0.01±0.00	0.12±0.05	0.02±0.01	0.23±0.13	0.79±0.09	0.02±0.01	0.11±0.07	0.24±0.06	0.03±0.01	0.05±0.01	0.02±0.00
oy104	0.00±0.00	0.12±0.05	0.36±0.07	0.29±0.04	0.00±0.00	0.00±0.00	0.05±0.02	0.00±0.00	0.39±0.20	0.65±0.18	0.05±0.04	0.38±0.14	0.66±0.09	0.01±0.01	0.03±0.02	0.01±0.00
oy105	0.08±0.03	0.22±0.09	0.18±0.10	0.25±0.10	0.10±0.02	0.15±0.06	0.29±0.16	0.05±0.03	0.31±0.17	0.39±0.16	0.33±0.17	0.44±0.12	0.58±0.15	0.09±0.03	0.38±0.13	0.24±0.11
oy106	0.02±0.02	0.02±0.01	0.04±0.01	0.20±0.08	0.10±0.09	0.13±0.10	0.31±0.19	0.03±0.02	0.42±0.07	0.80±0.04	0.02±0.01	0.17±0.08	0.54±0.18	0.03±0.01	0.14±0.06	0.39±0.14
oy107	0.00±0.00	0.13±0.06	0.23±0.06	0.34±0.12	0.02±0.02	0.16±0.06	0.41±0.09	0.02±0.01	0.29±0.15	0.70±0.13	0.19±0.06	0.41±0.06	0.60±0.08	0.06±0.02	0.17±0.02	0.10±0.03
oy108	0.03±0.01	0.15±0.07	0.10±0.04	0.29±0.11	0.04±0.02	0.07±0.06	0.33±0.22	0.13±0.09	0.20±0.09	0.54±0.15	0.02±0.01	0.35±0.17	0.38±0.16	0.00±0.00	0.11±0.08	0.03±0.02
oy109	0.04±0.03	0.02±0.02	0.02±0.01	0.04±0.02	0.02±0.01	0.02±0.00	0.04±0.01	0.06±0.04	0.03±0.01	0.07±0.03	0.02±0.01	0.03±0.01	0.10±0.04	0.02±0.01	0.03±0.00	0.01±0.00

[a]All strains, except WT and *srbc-64*; *srbc-66,* contain stably integrated copies of the *str-3*p*::gfp* fusion gene (*kyIs128*).
[b]Shown are the mean±SEM proportion of dauers formed for the given strains and conditions at 25°C. Data are from at least 3 biologically independent trials of 40-110 animals per assay, with at least 2 technical replicates in each trial.
[c]Ethanol was used as the diluent for all tested ascarosides compounds.
