# Supplementary material for: A Forward Genetic Screen for Molecules Involved in Pheromone-Induced Dauer Formation in Caenorhabditis elegans
Source: G3 (Bethesda). 2016 Mar 10;6(5):1475–87. doi: 10.1534/g3.115.026450 (PMC4856098; doi:10.1534/g3.115.026450)
Supplement: Supplemental Material [file supp_g3.115.026450_TableS3.pdf]

Table S3. Whole genome resequencing metrics.

Metric	Strain						
	oy103	*oy104*	*oy105*	*oy106*	*oy107*	*oy108*	*oy109*
Average genome coverage[a]	29.7X	33.2X	23.1X	23.3X	32.6X	33.8X	36.8X
Variants[b]	3659	3783	3783	3767	3735	3670	4441
Genic variants[c]	317	345	341	312	370	244	356
Unique coding variants[d]	102	118	129	97	148	31	132
Unique nonsense mutations[e]	4	3	8	3	3	0	3
Unique uncovered regions[f]	1	2	13	12	14	18	230

[a]Average depth of read coverage across unique regions in the genome.
[b]Total number of detected variants with respect to the N2 reference genome, including genic and intergenic regions. Only those variants that are supported by at least five overlapping consensus reads are reported in all cases.
[c]Variants in genic regions.
[d]Unique variants in coding regions present in individual genomes.
[e]Unique variants predicted to encode a premature termination codon in at least one isoform of a given locus in individual genomes.
[f]Genic regions lacking coverage in a given strain, but which are covered by reads in other sequenced strains.
