# Supplementary figures and images for: A Forward Genetic Screen for Molecules Involved in Pheromone-Induced Dauer Formation in Caenorhabditis elegans
Source: G3 (Bethesda). 2016 Mar 10;6(5):1475–87. doi: 10.1534/g3.115.026450 (PMC4856098; doi:10.1534/g3.115.026450)

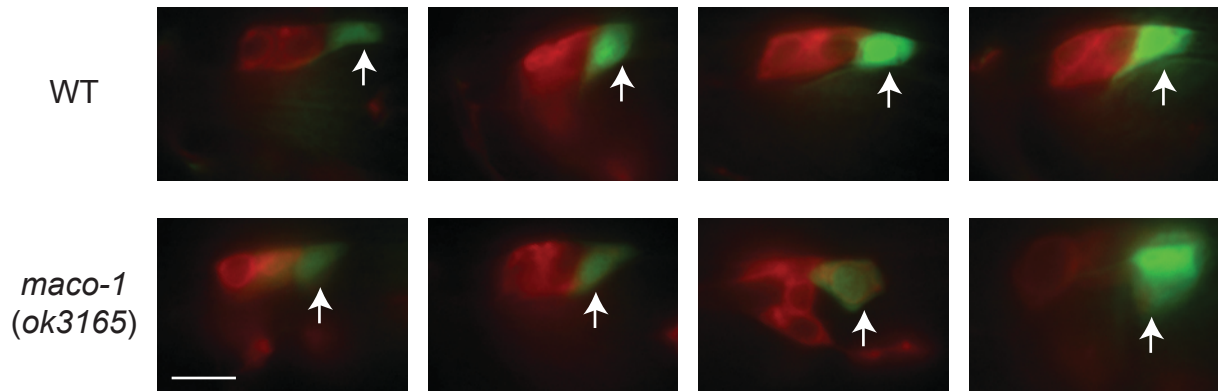

Supplement: Supplemental Material [file supp_g3.115.026450_FigureS1.ps]

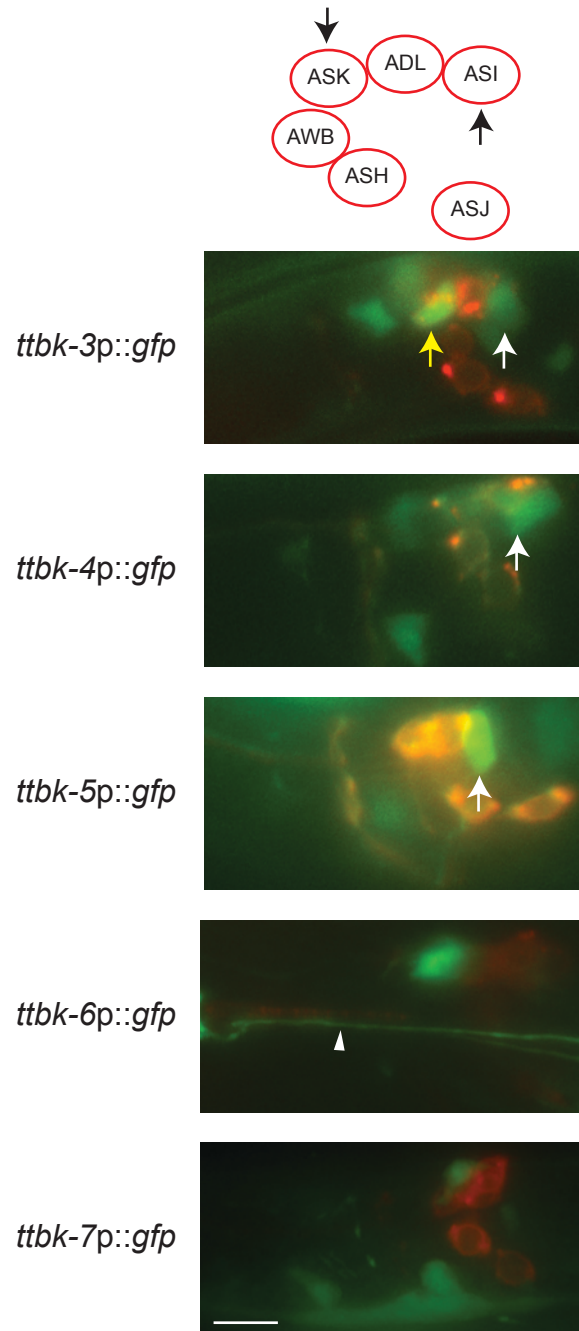

Supplement: Supplemental Material [file supp_g3.115.026450_FigureS2.ps]

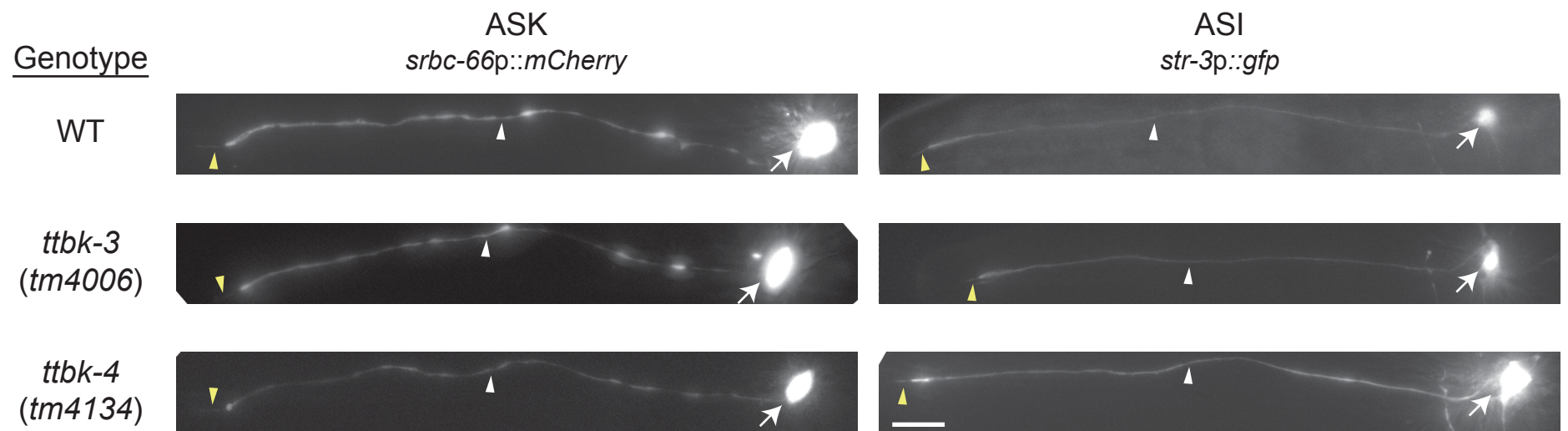

Supplement: Supplemental Material [file supp_g3.115.026450_FigureS3.ps]
